# Development of an iPSC-derived immunocompetent skin model for identification of skin sensitizing substances

**DOI:** 10.1177/20417314251336296

**Published:** 2025-05-06

**Authors:** Marla Dubau, Tarada Tripetchr, Lava Mahmoud, Fabian Schumacher, Burkhard Kleuser

**Affiliations:** 1 Freie Universität Berlin, Department of Pharmacology and Toxicology, Institute of Pharmacy, Berlin, Germany

**Keywords:** induced pluripotent stem cells, dendritic cells, immunocompetent skin model, skin sensitization, cytokine secretion, adverse outcome pathway, sphingolipids

## Abstract

The development of immunocompetent skin models marks a significant advancement in in vitro methods for detecting skin sensitizers while adhering to the 3R principles, which aim to reduce, refine, and replace animal testing. This study introduces for the first time an advanced immunocompetent skin model constructed entirely from induced pluripotent stem cell (iPSC)-derived cell types, including fibroblasts (iPSC-FB), keratinocytes (iPSC-KC), and fully integrated dendritic cells (iPSC-DC). To evaluate the skin model’s capacity, the model was treated topically with a range of well-characterized skin sensitizers varying in potency. The results indicate that the iPSC-derived immunocompetent skin model successfully replicates the physiological responses of human skin, offering a robust and reliable alternative to animal models for skin sensitization testing, allowing detection of extreme and even weak sensitizers. By addressing critical aspects of immune activation and cytokine signaling, this model provides an ethical, comprehensive tool for regulatory toxicology and dermatological research.

## Introduction

Skin sensitization is a complex immunological response initiated by exposure to specific chemical allergens, which ultimately results in the development of allergic contact dermatitis.^1^ The accurate identification of sensitizing substances is a critical requirement in the safety evaluation of chemicals, cosmetics, and pharmaceuticals. Despite the essential role of skin sensitization assays, the current methodologies face significant constraints that necessitate the development of more sophisticated, physiologically relevant models.^2^

Traditional animal-based assays, including the guinea pig tests (GPMT) (OECD TG 406)^3^ and the murine Local Lymph Node Assay (LLNA: OECD TG 429, 442A,B),^4–6^ have historically served as standard methods for assessing skin sensitization. However, these methods are facing increasing challenges from an ethical standpoint, regulatory restrictions, and limitations in their translational applicability due to the existence of inherent interspecies differences. Their limited accuracy, approximately 75% for the LLNA^7^ in predicting human sensitization potential, further emphasizes the need for alternative approaches that better reflect human biology.

Moreover, the reliance on animal models is inconsistent with the positions of the Organization for Economic Co-operation and Development (OECD), summarized in the Adverse Outcome Pathway (AOP) framework for skin sensitization, which aims to map the series of key events leading to an adverse outcome in humans.^8,9^

The skin sensitization AOP contains four key events. The first key event involves the covalent binding of sensitizing chemicals to skin proteins, a process called haptenation, forming hapten-protein complexes recognized as foreign by the immune system.^8^ Following this, in key event 2, keratinocytes become activated and release a variety of pro-inflammatory cytokines such as interleukin-1β (IL-1β), tumor necrosis factor-alpha (TNF-α), and interleukin-18 (IL-18), along with chemokines like CCL4 (MIP-1β) and CXCL8 (IL-8).^10,11^ These signaling molecules recruit immune cells to the site of exposure, creating an inflammatory environment that initiates the immune response.^10,11^

In key event 3, dendritic cells, particularly Langerhans cells in the skin, respond to cytokines and direct exposure to sensitizers by maturing and migrating to lymph nodes. During this activation, dendritic cells upregulate surface markers such as CD86, CD80, and CD40, which are essential for effective T-cell activation, and secrete cytokines such as IL-18 to drive Th1 differentiation and interleukin-10 (IL-10) to modulate the inflammatory response.^12–14^ They also increase the expression of MHC class II molecules, which present processed antigens to naive T cells. Finally, in key event 4, these antigen-presenting dendritic cells interact with naive T cells in the lymph nodes, leading to T-cell activation and clonal expansion, ultimately resulting in the development of sensitization. These coordinated immune events underline the complex yet efficient mechanisms of the skin’s response to sensitizing agents.

In light of the limitations of animal-based testing, in vitro approaches have emerged as a prominent alternative. The objective of these methods is to circumvent the ethical constraints inherent to animal-based testing while simultaneously providing scalable and reproducible platforms for sensitization assessment. Many of these in vitro assays focus on individual key events within the AOP for skin sensitization, such as protein reactivity (key event 1), keratinocyte activation (key event 2), or dendritic cell activation (key event 3). Assays like the Direct Peptide Reactivity Assay (DPRA), KeratinoSens™, and Human Cell Line Activation Test (h-CLAT) are recognized by the OECD for addressing these events. However, these assays address only specific molecular or cellular steps and fail to capture the entirety of the sensitization process, which involves a cascade of interactions culminating in T-cell activation.^9^ Consequently, multiple assays must be combined to approximate the full AOP, posing challenges in terms of resource allocation and result interpretation.

A significant advancement in non-animal testing strategies is the 2-out-of-3 Defined Approach (2o3 DA),^15^ which improves the predictive accuracy of skin sensitization testing by combining multiple assay results. Under this framework, a substance is classified as a skin sensitizer if at least two out of three assays yield a positive result. The most commonly used combination includes Direct Peptide Reactivity Assay (DPRA), an in chemico assay assessing haptenation (key event 1); KeratinoSens™, a keratinocyte activation assay evaluating oxidative stress responses (key event 2); and h-CLAT (human Cell Line Activation Test), a dendritic cell activation assay measuring immune cell activation (key event 3).^15^ By integrating data from multiple key events, the 2o3 DA approach reduces false-positive and false-negative classifications, increasing the reliability of hazard identification compared to individual assays.^16^ This method is also aligned with regulatory requirements, allowing in vivo testing to be waived in certain cases if sufficient evidence from non-animal methods is provided. Advancements in in vitro assays targeting key event 2 and key event 3 have played a crucial role in improving non-animal approaches for skin sensitization assessment. These assays offer mechanistic insights into cellular responses, support regulatory decision-making, and contribute to the reduction of animal testing. However, no single in vitro assay fully captures the complexity of the sensitization process, making their integration into Defined Approaches, such as the 2o3 DA, essential for achieving accurate and comprehensive risk assessments. Despite these advancements, most of these approaches still lack the cellular complexity and immune integration found in human skin, reinforcing the need for fully immunocompetent 3D models capable of addressing multiple key events of the AOP simultaneously.

Two-dimensional (2D) monocultures of keratinocytes and fibroblasts, respectively lack the structural and cellular complexity of native skin^17^ and frequently exclude immune components, such as dendritic cells, which are critical for mediating interactions between keratinocytes and T cells. Reconstructed human skin (RHS) models offer enhanced structural relevance; however, their inability to fully integrate immune cell types limits their capacity to replicate the dynamic crosstalk necessary for assessing immune responses in skin sensitization.^18,19^

In order to address these limitations, there has been a growing interest in the development of immunocompetent skin models that integrate both the structural and immune components of human skin. Unlike single in vitro methods, 3D constructs recapitulate the layered architecture of human skin, including the dermis and epidermis, and provide a more accurate representation of structural and functional properties.^17^ The epidermis, composed primarily of keratinocytes, acts as the primary barrier against environmental insults, while the dermis, rich in fibroblasts and extracellular matrix, provides structural support and facilitates signaling between cells. These layers interact dynamically to maintain homeostasis and mediate immune responses.^20^ To enhance the immune relevance of 3D skin models, various studies have incorporated immune cells, such as dendritic cells and Langerhans cells into these constructs.^21,22^ For instance Kosten et al.^23^ developed an immunocompetent skin model by integrating MUTZ-3-derived Langerhans cells into a 3D skin equivalent, allowing for improved immune cell interaction with keratinocytes. Similarly, Bock et al.^14^ incorporated MUTZ-LC into the epidermal layer of a 3D skin model, demonstrating enhanced immune responses upon exposure to sensitizers. More recently, Hölken et al.^22,24^ explored the integration of THP-1-derived dendritic cells into 3D skin equivalents, further demonstrating the potential of immunocompetent models for applications in toxicology and dermatology. Additionally, Böttcher et al.^25^ examined the integration of MUTZ-Langerhans cells into a 3D full-thickness skin equivalent and analyzed the effects of serum reduction and undefined medium supplements on their differentiation. These advancements highlight the importance of full immune cell integration in 3D skin constructs to better replicate human skin immunity and sensitization responses. However, most published skin models utilize a co-culture approach where monocyte-derived dendritic cells (MoDC) or monocyte-derived Langerhans cells (MoLC) are only partially incorporated into the three-dimensional structure.^26,27^ Those models often show an activation of dendritic cells even in the absence of skin sensitizers. Therefore, typically, these immune cells are embedded in a gel and added between the dermal and epidermal layers during the later stages of model assembly.^26^ This method limits their integration with the surrounding tissue and diminishes the model’s ability to accurately replicate the complex immune interactions of human skin.^26,28^

Sphingolipids, key components of the epidermal lipid matrix, play a vital role in maintaining the structural integrity and barrier function of the skin. As essential elements of the stratum corneum, sphingolipids regulate keratinocyte differentiation, immune signaling, and inflammation, processes that are directly implicated in skin sensitization.^29^ Variations in sphingolipid composition influence skin barrier permeability and immune responses, underscoring the importance of accurately representing sphingolipid dynamics in advanced skin models.^29,30^ Furthermore, profiling these sphingolipids has been shown to distinguish between cell types, such as adipose tissues, emphasizing their potential as biomarkers for cellular identity and function.^31,32^ This highlights the potential of sphingolipid profiling as a tool for advancing the understanding of cell type-specific characteristics in both research and clinical contexts.

This study builds on these advancements by developing and characterizing a fully iPSC-derived immunocompetent skin model. The model integrates fibroblasts (iPSC-FB), keratinocytes (iPSC-KC), and dendritic cells (iPSC-DC) into a 3D construct, recapitulating the layered architecture and immune competence of human skin. In contrast to established skin models, iPSC-DC can be fully integrated into the immunocompetent skin model without activation. Activation of iPSC-DC can however be achieved after treatment with skin sensitizers. The dermal layer, composed of iPSC-FB suspended in collagen, provides structural support, while the stratified epidermal layer of iPSC-KC forms a functional barrier. Integrated iPSC-DC replicate the antigen-presenting and immune-activating functions of primary dendritic cells, enabling the model to address multiple key events within the AOP for skin sensitization.

By capturing the complexity of skin structure and immune interactions, this iPSC-derived immunocompetent skin model represents a significant advancement in dermatological and toxicological research. It offers a robust, human-relevant platform for assessing skin sensitizers, addressing critical gaps in existing methodologies while aligning with regulatory shifts and the 3R principles. Through a comprehensive characterization of the model’s structural and immunological properties, this study demonstrates the skin model’s potential as a comprehensive, ethical alternative to traditional animal-based and isolated in vitro methods for skin sensitization testing.

## Materials and methods

### Hair follicle isolation

Human hair follicle-derived keratinocytes (HFDK) and fibroblasts (HFDF) were isolated following the previously described method of Löwa et al.,^20^ as detailed in our recent study.^33^ Briefly, 30–40 hair follicles were plucked from male and female donors (aged 25–35 years, ethical approval EA2/006/15) and placed onto poly-D-lysine-coated transwell inserts. Postmitotic 3T3-J2 fibroblasts were used as feeder cells, and the follicles were cultured in outer root sheath (ORS) medium until 70% confluency was reached. After 3 weeks, the outgrown cells were harvested by selective trypsinization to obtain HFDF and HFDK. HFDF were further cultured in fibroblast growth medium (FGM) containing DMEM supplemented with 10% fetal bovine serum (FBS), 1% *L*-glutamine, and 1% penicillin/streptomycin (the medium and supplements were from Sigma-Aldrich, Munich, Germany), while HFDK were maintained in keratinocyte growth medium 2 (KGM2;)PromoCell, Heidelberg, Germany).

### Reprogramming HFDK into iPSC

HFDK were reprogrammed into induced pluripotent stem cells (iPSC) using the CytoTune™-iPS 2.0 Sendai Reprogramming Kit (ThermoFisher Scientific, Waltham, USA) as described.^33^ Briefly, HFDK were cultured in KGM-2 medium and transduced with Sendai viral vectors containing hOct3/4, hSox2, hKlf4, and hc-Myc at a multiplicity of infection (MOI) of 3.6:3.6:2, according to the manufacturer’s instructions. Cells were subsequently cultured on vitronectin-coated plates in Essential 8™ medium (ThermoFisher Scientific, Karlsruhe, Germany) until fully reprogrammed iPSC colonies with typical morphology were selected.

### Characterization of iPSC and EB through histology and immunofluorescence staining

1x10^5^ iPSC were seeded into one-chamber slides (Sarstedt, Nümbrecht, Germany) and cultured in TeSR™-E8™ medium (Stemcell Technologies, Lyon, France) on vitronectin-coated chamber slides for 48 h prior to immunofluorescence staining. Immunofluorescence staining was performed according to our previous study.^33^ iPSC were incubated overnight with the following primary antibodies: anti-Ms X SSEA-4, Rb X Nano, human (both from Merck, Darmstadt, Germany), anti-TRA-1-60 Monoclonal Antibody (ThermoFisher Scientific, Karlsruhe, Germany), Sox2 Antibody, anti-human/mouse, REAfinity™ or Oct3/4 Isoform A Antibody, anti-human/mouse, APC, REAfinity™ (both from Miltenyi Biotec GmbH, Bergisch Gladbach, Germany). All antibody dilutions were prepared according to the manufacturer’s instructions. Alkaline phosphatase staining of the iPSC-colonies was performed with Alkaline Phosphatase Live Stain (500X) (ThermoFisher Scientific, Karlsruhe, Germany), according to the manufacturer’s instructions.

Embryoid bodies (EB) were generated as described.^33^ After their collection from hanging drops, EB were cultured in TeSR™-E8™ medium for 5 days. They were then embedded in Frozen Section Compound (Leica Biosystems, Wetzlar, Germany) in a 24-well cryotray, snap-frozen in liquid nitrogen, and stored at −80°C. EB sections were prepared by vertically cutting frozen samples into 7 µm-thick slices using a Leica CM1510 Cryotome (Leica Biosystems, Wetzlar, Germany). Sections were placed onto Poly-Prep microscope slides (Merck, Darmstadt, Germany). For hematoxylin and eosin (H&E) staining, EB sections were fixed using 4% Roti-Histo-Fix (Carl Roth, Karlsruhe, Germany) and stained following standard protocols (materials from Carl Roth, Karlsruhe, Germany). For immunofluorescence staining, EB were permeabilized with 0.5% (v/v) Triton-X in PBS for 15 min after the initial fixation. The staining protocol for iPSC was then applied to the EB sections. EB were stained with primary antibodies against NanoG and TRA-1-60, followed by the appropriate secondary antibodies. Slides were mounted with ProLong™ Gold antifade mountant with DAPI (4′,6-diamidino-2-phenylindole; ThermoFisher Scientific, Karlsruhe, Germany) and analyzed using fluorescence microscopy. Pictures were taken with a KZ-8001 Fluorescence microscope (Keyence, Neu-Isenburg, Germany).

### Evaluation of senescence in iPSC

To evaluate cellular senescence in iPSC, cells were prepared using a standardized protocol optimized for flow cytometry (CellEvent™ Senescence Green Flow Cytometry Assay Kit, ThermoFisher Scientific, Karlsruhe, Germany). iPSC incubated in CellEvent™ Senescence buffer devoid of any senescence-inducing agent served as a negative control. iPSC from passages 5 to 25 were collected and resuspended in TeSR™-E8™ medium at a concentration of 1 × 10^6^ cells per mL. Following collection, the cells were subjected to centrifugation, after which the resulting pellets were resuspended in 400 µL of 2% paraformaldehyde fixation solution and incubated on ice for 15 min in the dark. Subsequently, cells were subjected to centrifugation and washing with 800 µL of cell staining buffer (BioLegend, San Diego, USA), to remove the fixation solution. The cell pellets were then resuspended in senescence detection working solution (provided in the kit), whereas the negative control cells were resuspended in CellEvent™ Senescence buffer and incubated for 1 h at 37°C, protected from light and without CO_2_ exposure. Following the incubation period, the cells were subjected to centrifugation, washing with cell staining buffer and subsequently resuspended in 300 µL of cell staining buffer and analyzed by flow cytometry with a CytoFLEX flow cytometer (Beckman Coulter, Krefeld, Germany).

### Assessment of iPSC differentiation into iPSC-FB and iPSC-KC via sphingolipid profiling

To assess the successful differentiation of iPSC into keratinocytes and fibroblasts, the sphingolipid profiles of iPSC-derived cells were compared with those of primary keratinocytes and fibroblasts isolated from different sources (foreskin (isolated as previously described)^33^ and hair follicle). In addition, the sphingolipid composition of undifferentiated iPSC was analyzed to confirm that differentiation results in distinct, cell type-specific sphingolipid profiles. Normal human epidermal keratinocytes (NHDK) and HFDK were cultured in KGM-2 medium. iPSC-KC were maintained in CnT-Prime Epithelial Proliferation medium (CELLnTEC, Bern, Switzerland), while undifferentiated iPSC were cultured in TeSR™-E8™ medium. Normal human dermal fibroblasts (NHDF), HFDF and iPSC-FB were cultured in FGM. To standardize conditions and minimize external lipid contributions, fibroblasts were transferred to CnT-Prime Fibroblast ECM medium (CELLnTEC, Bern, Switzerland), a serum-free medium, 24 h before the experiment. This adaptation was not necessary for keratinocytes and iPSC as their respective culture media were already serum-free.

For sphingolipid analysis, 0.5 × 10^6^ cells per cell type were seeded onto 6 cm cell culture dishes and cultured in their respective growth media for 24 h. Cells were harvested and subjected to lipid extraction using 1.5 mL methanol/chloroform 2:1 (v/v) as described.^34^ The extraction solvent contained C17:0 ceramide (C17:0 Cer), d_31_-C16:0 sphingomyelin (d_31_-C16:0 SM), d_7_-sphingosine (d_7_-Sph), d_7_-dihydrosphingosine (d_7_-dhSph) and d_7_-sphingosine 1-phosphate (d_7_-S1P) as internal standards (all from Avanti Polar Lipids, Alabaster, USA). Sphingolipid species were analyzed by high-performance liquid chromatography coupled to electrospray ionization tandem-mass spectrometry (HPLC-ESI-MS/MS). Briefly, sphingolipid subspecies were chromatographically separated using a 1290 Infinity II HPLC system (Agilent Technologies, Waldbronn, Germany) equipped with a Poroshell 120 EC-C8 column (3.0 × 150 mm, 2.7 μm) and a matching guard column (3.0 × 5 mm, 2.7 μm; both Agilent Technologies). The HPLC eluate was diverted into an electrospray ion source operating in positive ionization mode (ESI+). Individual sphingolipid species were analyzed by multiple reaction monitoring (MRM) using a 6495C triple-quadrupole mass spectrometer (Agilent Technologies). Settings of the ESI source and MS/MS detector have been published recently.^34^ Peak areas of ceramide (Cer), dihydroceramide (dhCer), sphingomyelin (SM), and dihydrosphingomyelin (dhSM) subspecies were normalized to those of their internal standards (C17:0 Cer for Cer and dhCer subspecies; d_31_-C16:0 SM for SM and dhSM subspecies) followed by external calibration. DhSph, Sph, and S1P were directly quantified via their deuterated internal standards d_7_-dhSph, d_7_-Sph and d_7_-S1P, respectively. Data analysis was performed using MassHunter Quantitative Analysis software (version 10.1, Agilent Technologies).

### Differentiation of iPSC into CD14^+^ monocytes and dendritic cells

For the differentiation of iPSC into dendritic cells, the iPSC were first cultured on vitronectin-coated 6-well plates until they reached approximately 70% confluency. Subsequently, the cells were transferred to Geltrex™-coated plates (Geltrex™ LDEV-Free, hESC-Qualified, Reduced Growth Factor Basement Membrane Matrix, ThermoFisher, Karlsruhe, Germany). The passaging of iPSC was conducted using 0.5 mM EDTA in PBS (both from Sigma-Aldrich, Munich, Germany). For differentiation into CD14^+^ monocytes, the STEMdiff™ Monocyte Kit (StemCell Technologies, Lyon, France) was employed in accordance with the manufacturer’s instructions. Once the iPSC had reached a density of approximately 60 colonies per well on the Geltrex-coated 6-well plates, mesodermal differentiation was initiated by the addition of 2.5 mL of Medium A (prepared in accordance with the instructions provided with the kit) to each well. The plates were incubated at 37°C with 5% CO_2_. Following a 48-h incubation period, half of the medium was aspirated and replaced with 1.25 mL of fresh Medium A, and the plates were incubated for a further 24 h. To induce hematopoietic differentiation, on day 3, the medium was completely replaced with 2.5 mL of Medium B. After 48 h, half of the medium was again replaced with 1.25 mL of fresh Medium B. On day 7, Monocyte Differentiation Medium (prepared according to the manufacturer’s instructions) was added to each well, completely replacing the previous medium. Subsequently, the medium was replaced every other day for a period of 7 days. From day 14 onwards, an increase in floating cells was observed, indicative of monocyte maturation. The medium containing the floating cells was collected and subjected to centrifugation at 300*g* for a period of 10 min. The attached cells were maintained in Monocyte Differentiation Medium until day 30. To isolate CD14^+^ monocytes, the cell suspension was counted and resuspended in 80 µL of MACS BSA Stock Solution diluted 1:20 in autoMACS rinsing solution (both from Miltenyi Biotec, Bergisch Gladbach, Germany) per 10⁷ cells. Subsequently, 20 µL of CD14 MicroBeads, human (Miltenyi Biotec, Bergisch Gladbach, Germany) were added and incubated for 15 min in the dark at 4°C. Magnetic separation was conducted using a LS column, which was positioned within a MACS Separator (both from Miltenyi Biotec, Bergisch Gladbach, Germany). Subsequently, the column was rinsed with 3 mL of buffer, and the cell suspension was applied onto the column. The unlabeled cells were collected and the column was washed three times with 3 mL of buffer. The CD14^+^ cells were then eluted by removing the column from the magnetic field and flushing it with 5 mL of buffer using a plunger. In order to further differentiate CD14^+^ monocytes into iPSC-DC, 1 × 10^6^ monocytes were seeded into a single well of a 6-well plate containing 5 mL of ImmunoCult™-ACF Dendritic Cell Medium (StemCell Technologies, Lyon, France), supplemented with 50 µL of ImmunoCult™-ACF Dendritic Cell Differentiation Supplement. The cells were incubated at 37°C for a period of 3 days. On the third day, the medium was collected into a 15 mL Falcon tube and centrifuged at 300*g* for 10 min. The resulting cell pellet was resuspended in 5 mL of fresh ImmunoCult™-ACF Dendritic Cell Medium, supplemented with 50 µL of ImmunoCult™-ACF Dendritic Cell Differentiation Supplement. By day 5, the cells had fully differentiated into iPSC-DC and were ready for incorporation into the immunocompetent skin model.

### Flow cytometry analysis of the cells

HFDK, iPSC, and iPSC-DC were harvested and resuspended in FACS tubes at a concentration of 2 × 10^5^ cells per tube. The cells were washed twice with Cell Staining Buffer (BioLegend, San Diego, USA) in order to remove any debris and to ensure optimal staining conditions. For the purpose of conducting a marker analysis, the iPSC and iPSC-DC cells were stained with antibodies directed against CD83, CD86, HLA-DR, CD11c, and CD209 (all obtained from BioLegend, San Diego, USA). In contrast, the HFDK and iPSC cells were stained with antibodies directed against CD29 and CD49f (both obtained from Miltenyi Biotec, Bergisch Gladbach, Germany) and antibodies directed against SSEA4 and TRA-1-60 (both obtained from BioLegend, San Diego, USA). The antibodies were diluted in accordance with the manufacturer’s instructions, and the cells were incubated with the respective markers for 30 min on ice in the dark to ensure the preservation of fluorescence integrity. Subsequently, any unbound antibodies were removed by washing, and marker expression was quantified using a CytoFLEX flow cytometer. In order to ensure the accuracy and precision of the results, unstained cells were included as negative controls, and UltraComp eBeads™ Compensation Beads (Thermo Fisher Scientific, Karlsruhe, Germany) were employed for compensation, allowing for the precise correction of spectral overlap. The data were acquired and analyzed using the FlowJo software (version 10.8.01; BD Bioscience, San Jose, USA) and the CytoFLEX platform.

### Cytokine secretion by iPSC-DC in comparison to iPSC

To evaluate the cytokine secretion potential of iPSC-DC in comparison to undifferentiated iPSC, an ELISA was performed for IL-8 detection. Both iPSC and iPSC-DC were treated for 24 h with a positive cytokine cocktail composed of 50 ng/mL tumor necrosis factor-alpha (TNF-α) and 50 ng/mL interleukin-1 beta (IL-1β) (both from Miltenyi Biotech, Bergisch Gladbach, Germany). Untreated iPSC and untreated iPSC-DC served as controls. Following the treatment period, the culture supernatants were collected and analyzed using a human IL-8 ELISA kit (BioLegend, San Diego, USA), according to the manufacturer’s instructions. Cytokine levels were quantified using a TECAN Infinite M200 Pro plate reader (Tecan Group Ltd., Männedorf, Switzerland) at 450 nm, and the results were compared to assess differences in IL-8 secretion between the two cell types.

### Protein extraction and western blot of iPSC and iPSC-DC

For the protein extraction, iPSC at passage 19 and iPSC-DC (cultured for 2 days in ImmunoCult™ ACF with maturation supplement) were harvested, and cell pellets containing 1 × 10^6^ cells were resuspended in 125 µL RIPA buffer. Protein content was quantified using the Bradford assay as previously described.^33^ For the western blot analysis, a total of 20 µg protein from each sample was separated by 10% SDS-PAGE (Bio-Rad, Munich, Germany) at 200 V for 1.25 h. The proteins were subsequently transferred onto nitrocellulose membranes and blocked with 5% BSA for 1 hour at room temperature. Membranes were incubated overnight at 4°C with the following primary antibodies: Lin28a (1:1000, BioLegend, San Diego, USA), CD209 (1:500, Invitrogen, Carlsbad, USA), and CD11c (1:1000, Abcam, Cambridge, UK). Following primary antibody incubation, membranes were washed with a TBS-T buffer (tris-buffered saline with Tween) and incubated for 1 h at room temperature with horseradish peroxidase (HRP)-conjugated secondary antibodies: anti-mouse (Cell Signaling Technology, Frankfurt/Main, Germany) for Lin28a and CD209, and anti-rabbit (Cell Signaling Technology, Frankfurt/Main, Germany) for CD11c. Protein bands were detected using an enhanced chemiluminescence (ECL) substrate (Bio-Rad, Munich, Germany) and visualized using the ChemiDoc MP Imaging System. β-actin was used as a loading control to normalize protein expression levels.

### T-cell isolation from buffy coat

Naïve CD4^+^ T-lymphocytes were isolated from peripheral blood mononuclear cells (PBMCs), which were obtained from anonymous buffy coats, using the Naïve CD4+ T Cell Isolation Kit II (human; Miltenyi Biotec, Bergisch Gladbach, Germany), according to the manufacturer’s protocol. Briefly, non-CD4^+^ cells were labeled with a biotin-conjugated antibody cocktail targeting unwanted cell populations. These labeled cells were subsequently magnetically labeled using a CD4^+^ T-cell MicroBead cocktail. High-purity isolation of CD4^+^ T-cells was achieved through negative selection by depleting the magnetically labeled non-target cells.

### Mixed lymphocyte reaction assay

To demonstrate that mature iPSC-DC, generated in ImmunoCult ACF, promote allogeneic T-cell proliferation in a mixed lymphocyte reaction (MLR) assay, T-cell proliferation was assessed using flow cytometry. The differentiation of CD14^+^ iPSC-derived monocytes into iPSC-DC was conducted in accordance with the previously described method. A total of 1.5 × 10^6^ monocytes were resuspended in 5 mL of ImmunoCult ACF supplemented with 50 µL of differentiation supplement in one well of a six-well plate. This day is counted as day 0. On day 5, the cells were distributed into three different wells of the six-well plate and treated for 24 h with either a positive control, 2,4 dinitrochlorobenzene (DNCB, Sigma Aldrich, Munich, Germany) or left untreated. Therefore, 4 mL ImmunoCult ACF were added to each well, for the positive control, a mix of IL-1β and TNF-α was added at a final concentration of 50 ng/mL TNF-α and 50 ng/mL IL-1β, for the well containing DNCB, a final concentration of 5 µM was used. On day 6, carboxyfluorescein succinimidyl ester (CFSE)-labeled T-cells were prepared using the CellTrace™ CFSE Cell Proliferation Kit for flow cytometry (Invitrogen, Oregon, USA). The CD4-positive T-cells had been previously isolated of buffy-coat and stored in liquid nitrogen. The T-cells were thawed, counted and resuspended in PBS to achieve a concentration of 2 × 10^6^ cells in 2 mL PBS. To this, 2 µL Cell Trace from the CFSE kit were added and the cells were incubated 20 min in the dark at room temperature. Subsequently, 10 mL of FGM were added and the cells were incubated for a further 5 min in the dark at room temperature. They were then centrifuged for 10 min at 300*g* at room temperature and resuspended in 12 mL ImmunoCult-XF T-cell Expansion Medium (StemCell Technologies, Lyon, France). For the preparation of the iPSC-DC-T-cell coculture, the iPSC-DC were collected in separate 15 mL falcon tubes with one tube designated for each treatment and centrifuged at 300*g* for 10 min at room temperature. 1 × 10^5^ CFSE-labeled T-cells and 4000 iPSC-DC were added into appropriate wells of a 12-well plate in a 1:25 ratio in 3 mL ImmunoCult-XF T-cell expansion Medium. A well containing only CFSE-labeled T-cells without iPSC-DC served as a negative control for the assay, a well containing labeled T-cells supplemented with 25 µL/mL medium ImmunoCult™ Human CD3/CD28 T-Cell Activator (StemCell Technologies, Lyon, France) served as a positive control. The coculture was maintained for a period of 5 days with a medium change on day 3. On the fifth day, T-cell proliferation was quantified through flow cytometry.

### Generation of immunocompetent skin models

To construct immunocompetent skin models, iPSC-FB were cultured in T75 cm² flasks using CnT-Prime Fibroblast ECM Medium. Upon reaching appropriate confluency, cells were detached using TrypLE™ Select (Thermo Fisher Scientific, Karlsruhe, Germany). A total of 3.75 × 10^4^ iPSC-FB (passages 4–6) were resuspended in 260 µL of FBS and 240 µL of collagen I (PureCol™ EZ Gel Solution, Sigma-Aldrich, Munich, Germany) per skin model. For each model, 500 µL of the cell suspension was added onto Falcon^®^ Permeable Support transwell inserts (8 µm Transparent PET Membrane, membrane diameter: 10.5 mm, cell growth area: 0.9 cm^2^) placed in a 12-well plate. The plates were incubated at 37°C without CO_2_ for 2 h to allow the dermal collagen layer to solidify. After the collagen layer solidified, 500 µL of DC Base Medium XF (without cytokines; PromoCell, Heidelberg, Germany) was carefully added on top of the gel, and 1000 µL of medium was added to the well surrounding the insert. The constructs were incubated at 37°C and 5% CO_2_ for 2 h. In the next step, iPSC-KC, previously cultured in T150 cm^2^ in CnT Prime Epithelial Proliferation Medium were detached using TrypLE™ Select. iPSC-KC, passages 8–10 were resuspended along with iPSC-DC in a ratio of 0.9 × 10^6^ iPSC-KC and 0.2 × 10^6^ iPSC-DC per skin model in 500 µL of DC Base Medium XF per skin model. The medium from the solidified collagen layers was carefully aspirated, and 500 µL of the iPSC-KC and iPSC-DC suspension was layered on top of each collagen construct. The models were incubated for 24 h at 37°C and 5% CO_2_. Following the incubation, an airlift was performed 24 h later, to promote keratinocyte differentiation by culturing the models at an air-liquid interface. The skin models were maintained for 7 days, with medium changes every other day, using 1000 µL of fresh DC Base Medium XF. On day 7, the skin models were treated with a series of skin sensitizers of varying potencies for 24 h to assess their skin sensitization potential.

### Immunocompetent skin model viability assessment

The viability of the immunocompetent skin models after treatment with skin sensitizers of varying potencies was determined using the 3-(4,5-dimethylthiazol-2-yl)-2,5-diphenyltetrazolium bromide (MTT) assay. In addition to an untreated skin model (UT), as well as skin models treated with a positive control (pos), glycerol (Gly; CAS: 56-81-5), DNCB (CAS: 97-00-7), *p*-phenylenediamine (PPD; CAS: 10106-50-3), isoeugenol (IG; CAS: 97-54-1), resorcinol (RN; CAS: 108-46-3), and a vehicle control (0.1% dimethyl sulfoxide (DMSO) in medium) and a positive control (100% DMSO; CAS: 67-68-5, purity >99.8%) were included. All chemicals were obtained from Sigma-Aldrich (Munich, Germany). A 5 mg/mL MTT stock solution was diluted 1:10 with DC Base Medium XF. The inserts and wells of the immunocompetent skin models were washed twice with PBS before adding 500 µL MTT solution to the inserts and 1000 µL to each well. The plates were incubated for 3 h at 37°C and 5% CO_2_. Afterwards, the MTT solution was aspirated, and 500 µL of 100% DMSO was added to the inserts and 1 mL to the wells. The plates were shaken for 5 min at 600 rpm, and the absorbance was measured at 570 nm using a plate reader.

### Skin sensitization assay

Topical treatment of immunocompetent skin models with 10 µL of various compounds, differing in their potential to cause skin sensitization, was conducted. The following compounds were used; 500 µM glycerol, 5 µM DNCB, 10 µM *p*-phenylenediamine, 250 µM resorcinol, and 300 µM isoeugenol. Furthermore, a positive control was included, comprising an inflammatory cytokine cocktail containing 50 ng/mL TNF-α and 50 ng/mL IL-1β. Stock solutions of each skin sensitizer were prepared using DMSO as a solvent and subsequently diluted 1:1000 in DC Generation Medium XF, resulting in a final treatment DMSO concentration of 0.1%. Unexposed immunocompetent skin models were employed as untreated controls. The concentrations of each sensitizer were selected based on previous studies.^28,35–37^ Following treatment, the skin models were incubated for 24 h at 37°C and 5% CO_2_.

### Maturation of iPSC-DC as a readout of skin sensitization

Following the 24 h treatment period, the cells that had migrated out of the immunocompetent skin model and into the lower chamber of the transwell were collected. The migrated cells were stained with a cocktail of extracellular markers, including anti-HLA-DR-PE, anti-CD209-APC-A700, anti-CD86-BV605, and 7AAD (all from Biolegend, San Diego, USA; 7AAD from StemCell Technologies, Lyon, France). All antibodies were diluted in accordance with the manufacturer’s instructions. The staining procedure entailed washing the migrated cells twice in Cells Staining Buffer prior to incubating them with the marker cocktail for 30 min in the dark on ice. Subsequently, the cells were washed twice with Cells Staining Buffer to remove any residual markers. Unstained cells served as an unstained control and UltraComp eBeads™ Compensation Beads (Thermo Fisher Scientific, Karlsruhe, Germany) were used to achieve a compensation. Marker expression was measured and analyzed using a CytoFLEX flow cytometer and FlowJo version 10.8.01 (BD Bioscience, San Jose, USA) and represented as the percentage of marker-positive cells that migrated out of the skin model into the transwell.

### Viability assessment of iPSC-DC

To assess cell viability, 5 µL of 7AAD was added to each sample (treated with either 500 µM glycerol, 5 µM DNCB, 10 µM *p*-phenylenediamine, 250 µM resorcinol, 300 µM isoeugenol, or a positive control or left untreated) prepared for flow cytometry. Samples were then measured using a CytoFLEX flow cytometer. Unstained cells were included as an unstained control to set baseline parameters, and UltraComp eBeads™ Compensation Beads were used to achieve a compensation. Marker expression was measured and analyzed using a CytoFLEX flow cytometer.

### Immunofluorescent staining of immunocompetent skin models

Upon completion of the 7-day cultivation period, the membrane with the immunocompetent skin-models was excised from the insert and embedded in frozen section compound in a 24-well cryotray. Thereafter, the samples were rapidly frozen in liquid nitrogen and stored at −80°C. The frozen skin models were sectioned vertically with a Leica CM1510 Cryotome at a thickness of 7 µm and placed onto Poly-Prep microscope slides. In the first step, the slides containing the skin model sections were fixed using 4% Roti-Histo-Fix in order to facilitate subsequent staining. Following fixation, the skin model sections were permeabilized for 15 min with 0.5% (v/v) Triton X-100 in PBS for IF-staining and blocked with 1:20 goat serum in PBS for 30 min to reduce non-specific antibody binding. The sections were incubated overnight at 4°C in a dark environment with the following antibodies: anti-cytokeratin 10-REAfinity™, anti-cytokeratin 14 REAfinity™, anti-vimentin (all three from Miltenyi Biotech, Bergisch Gladbach, Germany), anti-filaggrin, anti-claudin, or anti-involucrin (all three from Invitrogen, Carlsbad, USA), anti-CD209, anti-HLA-DR, anti-Dectin-1 (all three from Miltenyi Biotech, Bergisch Gladbach, Germany). The next day, the slides were incubated with a secondary antibody, either Goat Anti-Rabbit Alexa Fluor 555 or Mouse Anti-Goat Alexa Fluor 488, for 1 hour at room temperature in the dark. After washing with PBS/BSA/Tween, a drop of ProLongTM Gold Antifade Mountant with DAPI was added. Pictures were taken with a KZ-8001 Fluorescence microscope.

### Cytokine secretion of immunocompetent skin models after 24 h treatment with skin sensitizers

Immunocompetent skin models were topically treated with 5 µM DNCB, 10 µM *p*-phenylenediamine, 300 µM isoeugenol, 250 µM resorcinol, 500 µM glycerol, or a positive control (IL-1β and TNFα). The treated models were incubated for 24 h at 37°C and 5% CO_2_. An untreated control was included to establish baseline cytokine levels. For the analysis of IL-1β-secretion, the skin model treated with a positive control was treated with 5 µL lipopolysaccharide (ThermoFisher, Karlsruhe, Germany). After the treatment period, the secretion of IL-8, TGF-β1, macrophage inflammatory protein- 1β (MIP-1β), thymic stromal lymphopoietin (TSLP), matrix metalloproteinase-9 (MMP-9), IL-18 and IL-1β was assessed using ELISA kits (Legend Max Human MMP-9 ELISA kit, Human TSLP Legend Max ELISA Kit, Human IL-1β ELISA Max™ Deluxe Set, Legend Max™ Total TGF-β1 ELISA Kit, and Legend Max™ High Sensitivity Human IL-8 Kit, all from BioLegend, San Diego, USA; Human MIP-1β Instant ELISA Kit, Human IL-18 ELISA Kit from ThermoFisher, Karlsruhe, Germany). The ELISA assays were conducted according to the manufacturer’s instructions. For the IL-8, IL-1β, and MIP-1β, TSLP, MMP-9 and IL-18 assays, samples were used undiluted. For the TGF-β1 assay, samples were diluted 1:2 in assay buffer. Absorbance was measured using a plate reader at 450 nm within 15 min of adding the stop solution to all wells.

## Results

### Reprogramming of HFDK into iPSC

**Figure 1.**
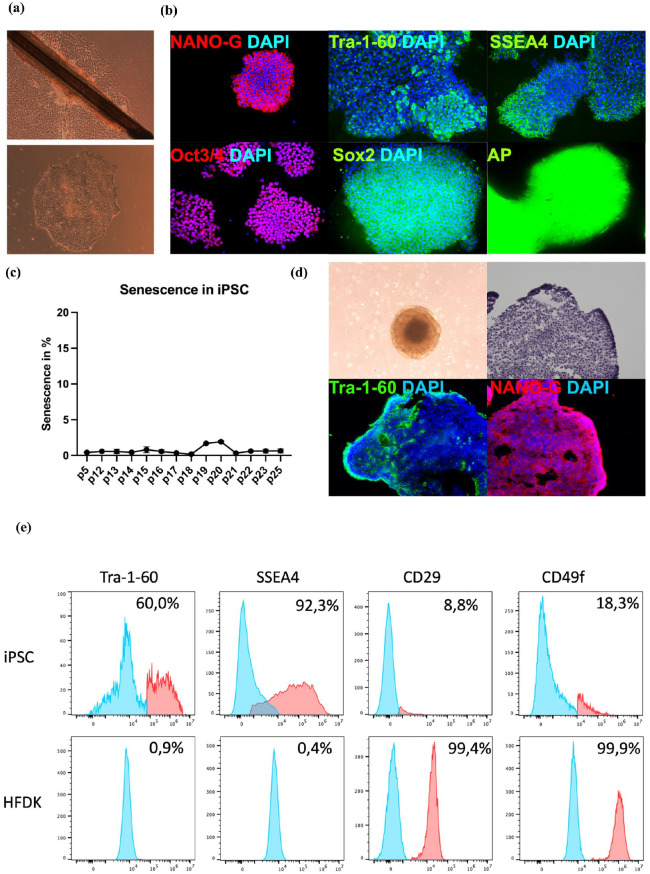
Pluripotency analysis of iPSC and embryoid bodies (EB). (a) phase-contrast microscopy images of cells outgrowing from a hair-follicle (top) and characteristic iPSC colony morphology with tightly packed cells (bottom). The pictures were taken at a magnification of 20×. (b) Immunofluorescence staining in iPSC against key pluripotency markers NANO-G, OCT3/4, So×2, Tra-1-60, and SSEA4, with nuclear counterstaining using DAPI. Alkaline phosphatase (AP) activity further validates the pluripotent state. The pictures were taken at magnification of 20× (*N* = 5). (c) iPSC from passages 5 to 25 were analyzed for cellular senescence using a fluorescent substrate specific for senescence-associated β-galactosidase activity, a key marker of senescence. The percentage of senescent cells was quantified and is presented as mean ± SD (*N* = 3). (d) Phase-contrast images depict EB formation, and histological staining confirms cellular organization within the EB. Immunostaining with Tra-1-60 (green) and NANOG (red) shows marker distribution within EB. The pictures were taken at a magnification of 20× (*N* = 3). (e) Flow cytometry analysis of surface markers. iPSC express high levels of pluripotency markers Tra-1-60 and SSEA4, while they exhibit lower levels of CD29 and CD49f. HFDK serve as negative control, showing minimal expression of Tra-1-60 and SSEA4 but high expression of CD29 and CD49f (*N* = 3).

To validate the successful reprogramming of HFDK into iPSC, flow cytometry analysis was performed to evaluate the expression of the pluripotency markers SSEA4 and Tra-1-60, as well as the keratinocyte-specific surface markers CD29 and CD49f. The analysis showed that CD29 and CD49f were highly expressed in HFDK, with over 99% of cells positive for these markers, whereas their expression was considerably reduced in iPSC. Conversely, the pluripotency markers SSEA4 and Tra-1-60 were expressed in less than 1% of HFDK, whereas iPSC showed robust expression of both markers. The loss of keratinocyte-specific surface marker expression and gaining of the iPSC-specific surface marker expression confirm the successful transition of HFDK to a pluripotent stem cell state (Figure 1(e)). Brightfield imaging (Figure 1(a)) provided further evidence, showing HFDK growing out of a hair follicle, which were used as the source for reprogramming into iPSC. The bottom panel of Figure 1(a) shows an established iPSC colony, demonstrating the distinct morphology of iPSC. Additionally, Figure 1(b) presents immunofluorescence staining of iPSC colonies, where the pluripotency markers Tra-1-60, NANO-G, SSEA4, Oct3/4, SOX2, and positive alkaline phosphatase (AP) staining were observed, consistent with our earlier findings published recently. These results, together with the flow cytometry data, further support the successful reprogramming of HFDK into a pluripotent stem cell state and complement our previous characterization.^33^

### Evaluation of senescence in iPSC

To assess the impact of increasing passage numbers on the process of senescence in iPSC the levels of senescence were measured using the CellEvent™ Senescence Assay, followed by flow cytometry. The study encompassed iPSC from earlier passages (passage 5) up to higher passages (passage 25). The findings indicate that, irrespective of the increase in passage number, the proportion of senescent iPSC remains consistently low. Even at higher passages, such as passage 25, the levels of senescence remained below 3%, with only slight variations observed between earlier passages and later ones (Figure 1(c)). These findings suggest that iPSC retain low levels of senescence throughout numerous passages, reflecting their robust capacity to maintain a proliferative, non-senescent state over extended periods of culture.

### Characterization of EB generated for differentiation of iPSC into FB and KC

To evaluate the quality and structure of embryoid bodies generated for differentiation of iPSC into fibroblasts and keratinocytes, a morphological and marker analysis of the EB was performed. Brightfield imaging of the EB revealed well-formed, spherical structures, characteristic of healthy EB. Hematoxylin and eosin staining showed organized cellular arrangements within the EB. IF-staining further confirmed the pluripotent nature of the EB, with robust expression of Tra-1-60 and NANO-G. These results confirm that the EB retained pluripotency and were morphologically suitable for directed differentiation into FB and KC lineages (Figure 1(d)).

### Characterization of iPSC-FB and iPSC-KC

In our recent study,^33^ the differentiation of iPSC into fibroblasts and keratinocytes were characterized using immunofluorescence staining and flow cytometry, which confirmed the expression of cell type-specific markers. Building on these findings, the current study aims to further investigate the differentiation process by analyzing the sphingolipid profiles of iPSC-derived cells in comparison to primary fibroblasts and keratinocytes. This additional lipidomic analysis provides deeper insights into the biochemical changes associated with cellular differentiation.

**Figure 2.**
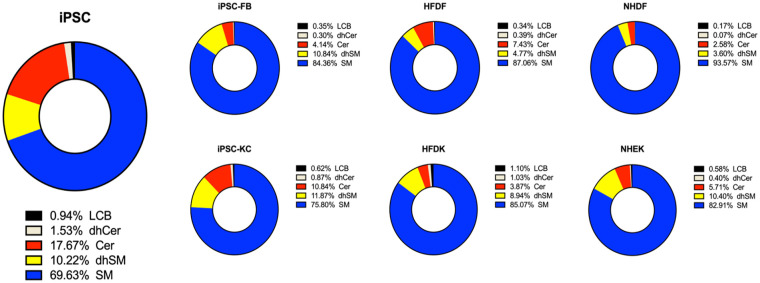
Quantitative analysis of sphingolipids in iPSC in comparison to hair-follicle, normal human foreskin-derived and iPSC-derived fibroblasts and keratinocytes. Levels of sphingolipid species, including ceramides (Cer), dihydroceramides (dhCer), sphingomyelins (SM), dihydrosphingomyelins (dhSM), and long-chain bases (LCB) in iPSC, iPSC-FB, HFDF, NHDF, iPSC-KC, HFDK, and NEHK summarized in pie charts.

The levels of ceramides were found to be comparable in all fibroblast types, including HFDF, NHDF and iPSC-FB, in terms of both the total ceramide content and the distribution of individual ceramide species. In contrast, undifferentiated iPSC exhibited a markedly elevated total ceramide concentration, accompanied by a pronounced enrichment in C16-C20 ceramides in comparison to fibroblasts (Figures 2 and S1). A comparable pattern was identified for dihydroceramides (dhCer), whereby total levels and C16-C20 species were elevated in iPSC compared to fibroblasts. The analysis of dihydrosphingomyelins (dhSM) showed remarkable differences. While total dhSM levels were lower in HFDF and NHDF compared to iPSC-FB and iPSC, specific subspecies showed unique patterns. Although iPSC and iPSC-FB show comparable total levels of dhSM, the subspecies distribution of iPSC-FB is more similar to that of HFDF and NHDF than to iPSC. Additionally, the total amount of sphingomyelins (SM) was slightly lower in both iPSC and iPSC-FB compared to HFDF and NHDF. However, the C18-C20 SM subspecies were significantly higher in iPSC than in all fibroblast types, whereas the C22-C24 SM subspecies were more abundant in all fibroblasts compared to iPSC. For long-chain bases (LCBs), including dihydrosphingosine (dhSph), sphingosine (Sph), and sphingosine 1-phosphate (S1P), levels were consistent across fibroblast types. However, iPSC had a significantly higher LCB concentration than fibroblasts, highlighting the unique sphingolipid composition of pluripotent cells (Figure 2). A more detailed overview of the individual sphingolipid species and their distribution is provided in the Supplemental Figure S1.

In keratinocytes, the sphingolipid profiles of NHEK, HFDK, iPSC-KC, and iPSC exhibited additional differences. The total ceramide levels were observed to be marginally elevated in iPSC-KC in comparison to NHEK and HFDK, with HFDK exhibiting a ceramide concentration that was approximately half that of NHEK (Figure 2). Conversely, the total ceramide levels in iPSC were observed to be 1.6-fold higher than those in iPSC-KC. With regard to dihydroceramides, total levels were found to be comparable between all keratinocyte types, with iPSC exhibiting an up to threefold increase in total dihydroceramide levels. Furthermore, the chain length distribution of dihydroceramides was found to be consistent among the various keratinocyte types, with C18 being significantly lower in all cases compared to that observed in iPSC (Figure S2). In the case of dihydrosphingomyelins, total levels were comparable in iPSC and all types of keratinocytes, however, C18 dhSM levels were around ten times higher in iPSC compared to keratinocyte types. Similarly, the total amount of sphingomyelins (SM) was found to be comparable between iPSC and all keratinocytes. LCB levels were similar in NHEK, HFDK, iPSC-KC, and iPSC (Figure 2). More detailed information regarding the individual sphingolipid species in keratinocytes and their distributions is provided in the Supplemental Figure S2.

Notably, the sphingolipid profiles of iPSC-KC were highly comparable to those of primary keratinocytes (NHEK and HFDK) and different from undifferentiated iPSC (Figure 2). Similarly, the sphingolipid profile of iPSC-FB closely resembled that of primary fibroblasts (HFDF and NHDF), further confirming successful differentiation. These findings highlight the ability of iPSC to reprogram into keratinocyte- and fibroblast-specific lipid profiles, reflecting their respective lineages and metabolic states.

### Characterization of iPSC-DC

To generate dendritic cells from iPSC, the differentiation protocol was modified to direct the cells toward the hematopoietic lineage. iPSC were cultured on Geltrex-coated plates in defined media, which induced differentiation into CD14^+^ monocytes. The CD14^+^ cells were subsequently isolated from the negative fraction using a magnetic cell separation system, whereby the CD14^+^ fraction was retained in the column for subsequent processing.

**Figure 3.**
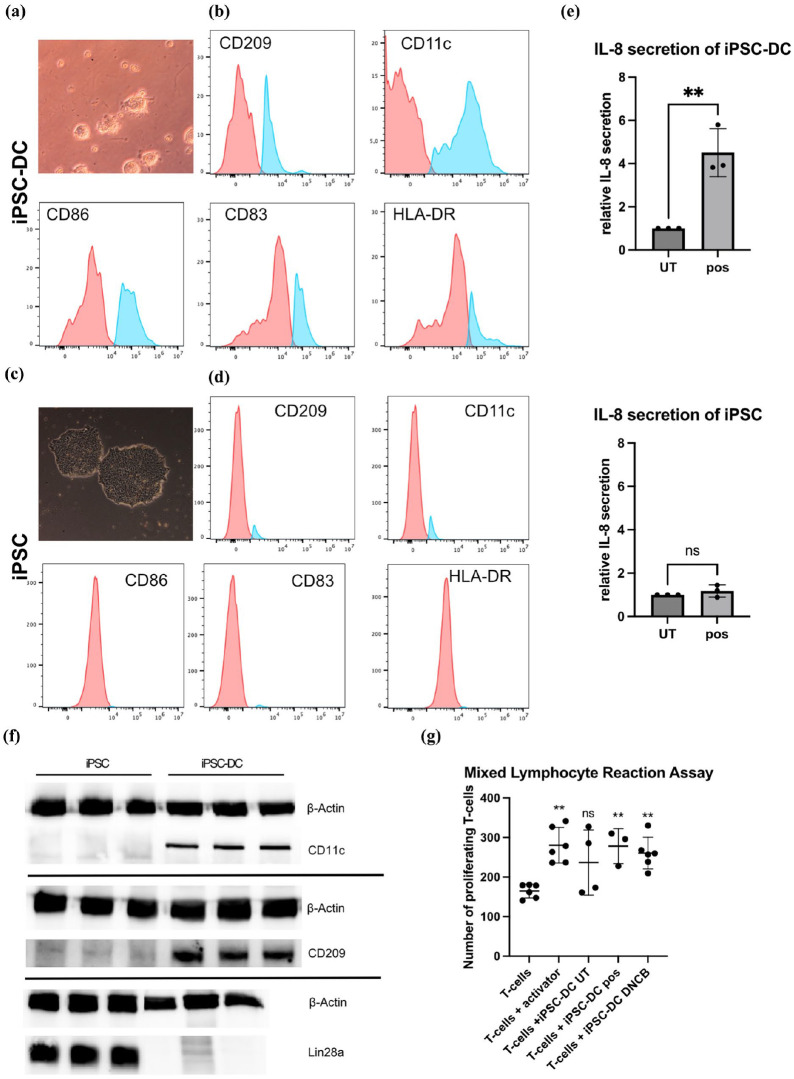
Functional and molecular characterization of iPSC-derived dendritic cells (iPSC-DC). (a and b) Phase-contrast microscopy images demonstrate distinct morphology. iPSC-DC display characteristic dendritic projections, while iPSC exhibit tightly packed colonies with a high nucleus-to-cytoplasm ratio and well-defined colony borders (magnification 20×). (c and d) iPSC-DC and iPSC were stained with fluorochrome-conjugated antibodies against HLA-DR, CD209, CD11c, CD86, and CD83. Unstained controls are shown in red histograms. (e) Relative IL-8 secretion of iPSC and iPSC-DC treated with a positive control composed of 50 ng/mL tumor necrosis factor-alpha (TNF-α) and 50 ng/mL interleukin-1 beta (IL-1β) in comparison to their untreated controls. (f) Western blot analysis of iPSC and iPSC-DC for the pluripotency marker Lin28a and the DC-markers CD11c and CD209, β-Actin was used as a loading control. (g) Mixed Lymphocyte Reaction Assay to assess antigen-presenting function of mature iPSC-DC: Mature iPSC-DC were co-cultured with 1 × 10 CFSE-labeled allogeneic CD4+ T cells at a DC:T cell ratio of 1:25. After 5 days of co-culture, proliferation of CD4+ CFSE-labeled T cells was analyzed by flow cytometry (*N* = 3–6). T cells cultured without DC, either with or without CD3/CD28 T cell activator, served as negative and positive controls, respectively. Mature DC treated with DNCB (positive control) significantly induced the proliferation of allogeneic T cells, demonstrating their functional antigen-presenting capability. Statistical analysis was performed using one-way ANOVA with Dunnett’s correction. **p-value ⩽ 0.01, ns: not significant. Data are presented as mean ± SD. (*N* = 3–6).

Following isolation, the monocytes were successfully differentiated into iPSC-DC through the addition of specific maturation supplements. To confirm successful differentiation, flow cytometry was performed to assess the expression of characteristic dendritic cell surface markers, including CD209, CD83, CD86, HLA-DR, and CD11c on iPSC-DC compared to iPSC. The flow cytometry results demonstrated high expression of these markers in iPSC-DC, while they were absent in the iPSC (Figure 3(b) and (d)), thereby confirming the successful differentiation of iPSC into iPSC-DC.

Western blot analysis provided further validation of these findings by comparing the expression of Lin28a, CD209, and CD11c between iPSC and iPSC-DC. iPSC expressed high levels of Lin28a, a key pluripotency marker, while showing no detectable expression of the dendritic cell markers CD11c and CD209. Conversely, iPSC-DC expressed high levels of CD11c and CD209, which are characteristic of mature dendritic cells. Additionally, there was an absence of Lin28a, indicating successful loss of pluripotency and acquisition of a dendritic cell phenotype (Figure 3(f)).

Furthermore, a microscopic image of an iPSC colony was included, displaying the typical morphology characterized by tightly packed cells forming dense, well-defined colonies with high nucleus to cytoplasm ratios and prominent nucleoli-features that reflect their pluripotent nature, alongside a microscopic image of iPSC-DC, highlighting their distinct dendrites and protrusions, hallmark features of mature dendritic cells (Figure 3(a) and (c)).

### Cytokine secretion by iPSC-DC in comparison to iPSC

To evaluate the cytokine secreting capacity of iPSC-DC an ELISA assay was performed. Both iPSC-DC and iPSC were treated with an inflammatory cytokine cocktail as a positive control or left untreated for a period of 24 h. A significant increase in IL-8 secretion was observed in iPSC-DC following stimulation with the positive control compared to untreated iPSC-DC. In contrast, no significant change in IL-8 levels was observed in iPSC irrespective of treatment. These results demonstrate that iPSC-DC, unlike iPSC, have the ability to secrete inflammatory cytokines in response to stimulation, underscoring their functional maturation (Figure 3(e)).

### Mixed lymphocyte reaction assay to assess antigen presentation by iPSC-DC

The mixed lymphocyte reaction (MLR) assay was employed to assess the antigen presenting capacity of mature iPSC-DC. T-cells cultured without iPSC-DC either without or with the ImmunoCult™ CD3/CD28 T-cell activator served as negative and positive controls. The findings revealed that T-cells cultured in the absence of both iPSC-DC and the T-cell activator demonstrated significantly diminished proliferation rates in comparison to those cultured in the presence of iPSC-DC or with the T-cell activator. In contrast, T-cells co-cultured with iPSC-DC demonstrated a marked increase in proliferation, indicating the effective stimulation by the iPSC-DC. The incubation of iPSC-DC for 24 h with either a positive control or 5 µM DNCB resulted in a notable enhancement of T-cell proliferation. In contrast, the untreated iPSC-DC demonstrated a tendency to promote T-cell proliferation, though to a lesser extent. Similarly, T-cells cultured with the T-cell activator served as a positive control and exhibited a robust proliferation (Figure 3(g)). Notably, stimulated iPSC-DC, pre-incubated with a positive control or 5 µM DNCB for 24 h, significantly enhanced T-cell proliferation compared to T-cells cultured alone. In contrast, unstimulated iPSC-DC failed to induce a statistically significant increase in T-cell proliferation, reflecting the typical behavior of dendritic cells in physiological settings, where activation is essential for robust T-cell stimulation.

### Development and characterization of immunocompetent skin models from iPSC-derived cells

In the next approach, full-thickness skin equivalents with an immune function were generated using iPSC-FB, iPSC-KC, and iPSC-DC. The initial step involved the resuspension of the iPSC-FB in a collagen I–FBS mixture and subsequent seeding onto a porous cell membrane. Subsequently, a four-hour incubation period was conducted to allow the dermal layer to solidify. During this time, the iPSC-KC and iPSC-DC were resuspended in xenofree DC-Base Medium and added on top of the dermal layer to form an epidermis. Following a 24-h period, the constructs were transitioned to an air-liquid interface, with the immunocompetent skin models then undergoing a further 7 days of culture.

**Figure 4.**
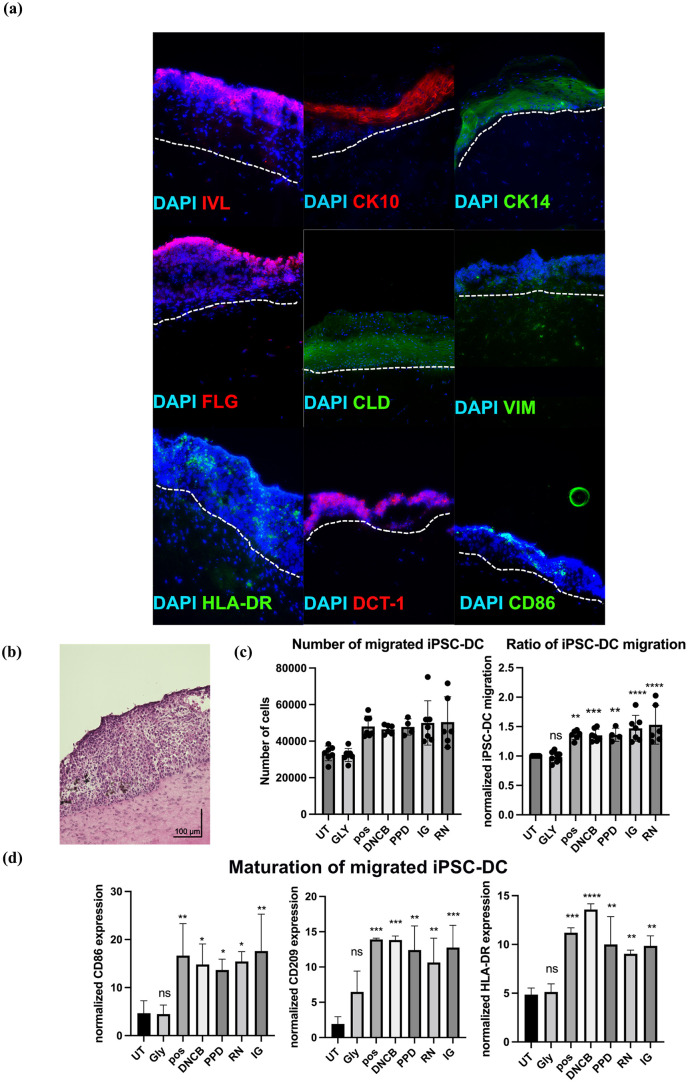
Characterization of immunocompetent skin models and assessment of iPSC-DC maturation after sensitization assay. (a) Immunofluorescence staining targeted epidermal markers including involucrin (IVL-red), cytokeratin 10 (CK10-red), and cytokeratin 14 (CK14-green). Tight junctions between cells were visualized using claudin (CLD-green) staining, and the dermal-epidermal junction was shown by filaggrin (FLG-red). The presence of iPSC-DC were marked by HLA-DR-green, Dectin-1 (DCT-1-red), CD86 (green). Cytoskeleton is indicated by vimentin with white arrows (VIM-green). Nuclei were counterstained with 4′,6-diamidino-2-phenylindole (DAPI). The pictures were taken at a magnification of 20×. The white dotted line indicates the epidermal-dermal interface. (b) H&E staining of cryosectioned immunocompetent skin model, with the scale bar indicating 100 µm. (c) Representation of iPSC-DC that migrated out of immunocompetent skin models after exposure to skin sensitizers, represented as total number of cells and migration ratio. (d) iPSC-DC that migrated out of the skin model were analyzed for maturation following the 24 h treatment with glycerol (Gly), 2,4 dinitrochlorobenzene (DNCB), p-phenylenediamine (PPD), isoeugenol (IG), resorcinol (RN), or a positive control, compared to untreated (UT) controls. Migrated cells were assessed by flow cytometry for the expression of key maturation markers, including CD86, HLA-DR, and CD209. Statistical significance determined by ordinary one-way ANOVA with Dunnett’s multiple comparison correction. (*N* = 3). *p-value ⩽ 0.05, **p-value ⩽ 0.001, ***p-value ⩽ 0.0001, ****p-value ⩽ 0.00001. Results are presented as mean ± SD.

The developed immunocompetent skin models were further characterized concerning their physiological structure and barrier functionality. Therefore, immunofluorescence staining was performed to verify the presence of tight junction proteins, including claudin, across the dermal and epidermal layers, thereby underscoring its pivotal role in regulating paracellular permeability. It is noteworthy that the expression of claudin was particularly pronounced within the epidermal layer of the immunocompetent skin models. Moreover, the skin models displayed the presence of filaggrin, an important protein involved in filament aggregation, which was predominantly expressed in the stratum corneum and situated at the outermost layer of the epidermis (Figure 4(a)). Additionally, the model displayed positive staining for CK10, indicative of the beginning stage of keratinocyte differentiation within the suprabasal layers of the epidermis. Furthermore, positive staining for CK14, indicative of the basal layer keratinocytes, serves to reinforce the notion of the skin model’s considerable cellular complexity. Involucrin is primarily expressed in the epidermis, specifically in the cells of the stratum spinosum and stratum granulosum, which constitute the outermost layers of the skin (Figure 4(a)). In the epidermal layer, the presence of the iPSC-DC has been observed to result in the expression of CD86, HLA-DR, and Dectin-1 (Figure 4(a)). Additionally, Figure 4(b) includes an image of the immunocompetent skin model after H&E staining, providing a general representation of its morphology.

### Maturation of migrated iPSC-DC following exposure to skin sensitizers

In order to investigate key event 3 of the AOP for skin sensitization, the maturation of migrated iPSC-DC were assessed following a 24-h exposure to a series of skin sensitizers of varying potencies and a non-sensitizer. Following the completion of the treatment period, the maturation rate of the migrated iPSC-DC were evaluated through flow cytometry. Subsequently, the medium from the transwells was collected, and the cells were stained for CD209, HLA-DR, and CD86 in order to characterize their activation and maturation status.

The analysis demonstrated that cells exposed to skin sensitizers, or the positive control exhibited significantly increased levels of CD209 and HLA-DR in comparison to the untreated controls and those treated with glycerol. This indicates that the immunocompetent skin model can effectively respond to sensitizers by promoting the migration and maturation of iPSC-DC from the skin model into the transwell system. Moreover, the expression of CD86, a key marker of dendritic cell maturation, was significantly increased in cells treated with skin sensitizers or the positive control in comparison to those treated with glycerol or left untreated (Figure 4(d)). Figure 4(c) presents the total number of iPSC-DC that migrated out of the immunocompetent skin model upon stimulation and the ratio of iPSC-DC treated with the non-sensitizer glycerol or the sensitizers DNCB, *p*-phenylenediamine, isoeugenol, resorcinol or the positive control, normalized to the untreated control, demonstrating a significant increase in cell migration following sensitizer treatment. These findings illustrate that the iPSC-DC within the skin model respond to sensitizer exposure by migrating out of the epidermal layer and maturing, thereby showing the model’s relevance for the assessment of skin sensitization events.

### Viability assessment of the immunocompetent skin model

**Figure 5.**
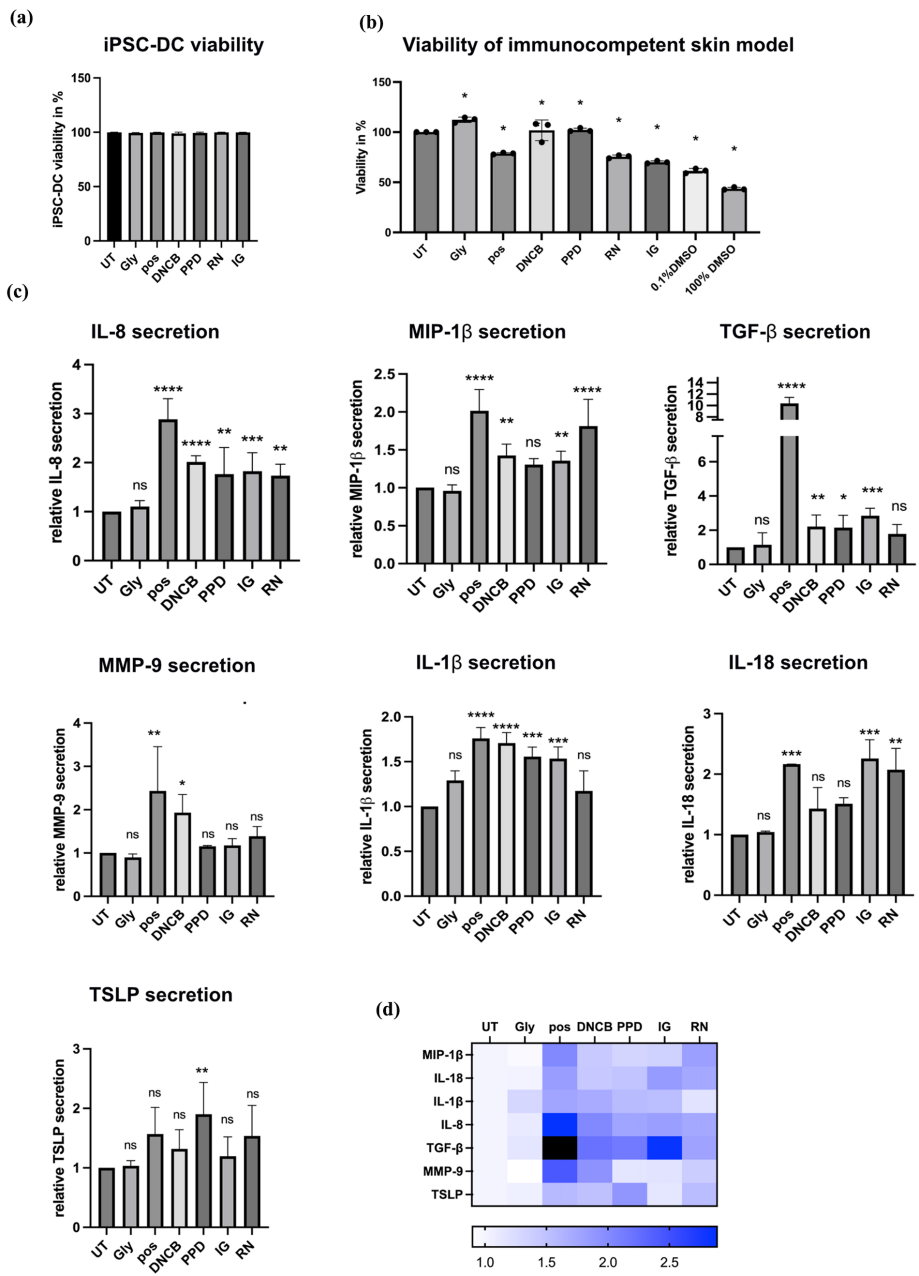
Assessment of viability and cytokine secretion in immunocompetent skin model. (a) DC-viability. Cell viability is determined by 7AAD. Data are presented as mean ± SD, (*N* = 3). *p ⩽ 0.05, ordinary one-way ANOVA with Dunnett’s correction for multiple comparisons. (b) Cell viability was determined via MTT assay, comparing untreated skin models (UT), a vehicle control (0.1% DMS0), a positive control (100% DMSO), and skin models treated with 500 µM glycerol (Gly), 5 µM 2,4 dinitrochlorobenzene (DNCB), 10 µM p-phenylenediamine (PPD), 300 µM isoeugenol (IG), or 250 µM resorcinol (RN). Data are presented as mean ± SD, (*N* = 3). *p ⩽ 0.0001, ordinary one-way ANOVA with Dunnett’s correction for multiple comparisons. (c) ELISA analysis for secretion of IL-8, MIP-1β, IL-1β, TGF-β, IL-18, MMP-9, and TSLP in immunocompetent skin models, that were exposed to Gly, pos, DNCB, PPD, IG, RN or left untreated for 24 h. Data are presented as mean ± SD, (*N* = 3). *p ⩽ 0.05, ordinary one-way ANOVA with Dunnett’s correction for multiple comparisons. (d) Heatmap displays the relative expression levels of cytokines (IL-8, MIP-1β, IL-1β, TGF-β, IL-18, MMP-9, and TSLP) in test groups subjected to different treatments: untreated (negative control), Gly, RN, DNCB, IG, PPD, and a positive control. Cytokine expression levels were normalized and color-coded, with dark blue representing higher expression, with light blue indicating lower expression, white denoting baseline levels, and black representing the out-of-range levels.

The overall viability of the cells included in the immunocompetent skin model was evaluated through the MTT assay. The skin models were treated with a range of sensitizers, including the extreme sensitizers DNCB and *p*-phenylenediamine, the moderate sensitizer isoeugenol, the weak sensitizer resorcinol, and glycerol as a non-sensitizing compound, all solubilized in DMSO. For purposes of comparative analysis, an untreated control and a positive control treated with an inflammatory cytokine cocktail were included. In the MTT assay, 100% DMSO was employed as a positive control to establish benchmarks for cell viability, while 0.1% DMSO was utilized as a vehicle control. The results demonstrated a robust viability across the untreated control, vehicle control, and skin sensitizer and non-sensitizer-treated samples (Figure 5(b)). This demonstrates that the skin models maintain a high metabolic activity over extended culture periods despite the iPSC-FB and iPSC-KC not being cultured in their respective medium, but in a dendritic-cell specific medium.

Furthermore, in order to assess the specific viability of the iPSC-DC, the viability of the iPSC-DC that had migrated through the skin model and membrane into the transwell was evaluated using flow cytometry analysis of 7-AAD staining. This allowed for the accurate determination of viable iPSC-DC populations within the transwell system. The results showed that the viability of iPSC-DC exceeded 98% across all treatment conditions (Figure 5(a)). This additional step confirmed that the iPSC-DC were viable after 7 days of co-culture with iPSC-FB and iPSC-KC, and that the observed effects were not due to cytotoxicity.

### Cytokine release of immunocompetent skin model following exposure to skin sensitizers

To evaluate cytokine release during skin sensitization, test substances with varying sensitizing potential were applied to iPSC-derived skin models containing dendritic cells, keratinocytes, and fibroblasts. The chosen test substances included the extreme sensitizers DNCB and *p*-phenylenediamine, the moderate sensitizer and pre-hapten isoeugenol, the weak sensitizer resorcinol, and the non-sensitizing compound glycerol. A pro-inflammatory cytokine cocktail was used as a positive control. Concentrations of the test substances were optimized to elicit functional responses relevant to sensitization while maintaining optimal cell viability. The cytokine analysis focused on mediators associated with key event 2, keratinocyte activation, and key event 3, dendritic cell activation and antigen presentation. The investigated cytokines included IL-8, IL-18, TGF-β, IL-1β, MIP-1β, MMP-9, and TSLP, which capture critical inflammatory and immune activation pathways in skin sensitization. Notably, IL-8 and IL-1β secretion plays a dual role, being linked to both KE2 and KE3, as they are secreted by both keratinocytes and dendritic cells during the sensitization process. After 24 h of topical treatment, significant upregulation of IL-8, IL-1β, MIP-1β, and TGF-β secretion was observed in the skin models treated with DNCB, *p*-phenylenediamine, and the positive control compared to untreated controls. The moderate sensitizer isoeugenol also induced a significant increase in the secretion of these cytokines, although to a lesser extent than the extreme sensitizers. In contrast, the weak sensitizer resorcinol showed a significant increase in IL-8 and MIP-1β secretion only, whereas the negative control glycerol did not induce any significant changes. For IL-18, significant increases in secretion were observed in skin models treated with the positive control, as well as with isoeugenol and resorcinol, compared to untreated controls. Although treatments with *p*-phenylenediamine and DNCB did not result in statistically significant increases, a clear trend toward higher IL-18 secretion was evident. No increase in IL-18 secretion was observed following treatment with the negative control, glycerol. To further address key event 2, cytokines primarily secreted by keratinocytes, such as MMP-9 and TSLP, were analyzed. MMP-9 secretion was significantly increased in the models treated with the positive control and DNCB, indicating keratinocytes activation. TSLP secretion showed a significant increase in models treated with *p*-phenylenediamine, with other sensitizers and the positive control displaying a clear upward trend that did not reach statistical significance. For key event 3, the secretion of cytokines primarily linked to dendritic cell activation, such as IL-18 and MIP-1β, demonstrated the model’s ability to capture dendritic cell-mediated immune responses. The robust secretion of MIP-1β and the shared role of IL-8 and IL-1β, secreted by both keratinocytes and dendritic cells, highlight the interconnectedness of key event 2 and key event 3 in the skin sensitization process (Figure 5(c) and (d)). These results demonstrate the ability of the iPSC-derived skin model to differentiate substances based on their sensitization potential, effectively addressing both key event 2 (keratinocyte activation) and key event 3 (dendritic cell activation) of the AOP for skin sensitization.

In summary, the present study successfully differentiated fibroblasts, keratinocytes, and dendritic cells from iPSC and incorporated them into a functional, immunocompetent skin model with fully integrated, non-activated dendritic cells. Upon exposure to skin sensitizers, the model demonstrated its immunological responsiveness by inducing dendritic cell migration through the skin model and maturation of the dendritic cells.

## Discussion

Many existing methods for assessing skin sensitization, such as the LLNA and GPMT, rely heavily on animal testing despite ethical concerns and increasing advocacy for alternative approaches. Modern in vitro assays, including the Direct Peptide Reactivity Assay (DPRA), KeratinoSens™, and Human Cell Line Activation Test (h-CLAT), are OECD^3,8,9,38^- recognized but focus on single key event within the AOP for skin sensitization. Similarly, in silico tools like QSAR (Quantitative Structure-Activity Relationship) models^2^ provide rapid, cost-effective predictions but struggle with complex chemicals and mixtures.

Several Defined Approaches have been developed to enhance skin sensitization testing by integrating New Approach Methodologies (NAMs), such as in vitro assays and in silico tools, to better mimic in vivo conditions. One widely accepted method is the 2o3 DA, which improves predictive accuracy by requiring that at least two out of three assays yield a positive result for a substance to be classified as a skin sensitizer.^15^ The most commonly used assays include DPRA, which assesses haptenation, KeratinoSens™, which evaluates oxidative stress responses, and h-CLAT, which measures immune cell activation.^15^ By combining data from multiple key events, the 2o3 DA enhances reliability compared to single assays and aligns with regulatory requirements, reducing the need for in vivo testing in certain cases. Despite these advancements, defined approaches still lack the biological complexity of human skin, particularly in capturing cell-cell interactions and immune responses. Many in vitro models do not fully integrate dendritic or Langerhans cells, limiting their ability to replicate the dynamic interplay between keratinocytes, fibroblasts, and immune cells in skin sensitization. Immunocompetent skin models have been proposed to overcome these limitations; however, models incorporating dendritic or Langerhans cells often fail to replicate the intricate interactions between skin and immune cells due to poor cell integration.

This study aimed to develop a fully integrated, iPSC-derived immunocompetent skin model capable of addressing multiple key events of the AOP simultaneously and detecting skin sensitizers of varying potencies, offering a comprehensive and physiologically relevant alternative and bridging the gap between NAM-based defined approaches and in vivo models.

iPSC were successfully differentiated into dendritic cells, confirmed by the absence of the pluripotency marker Lin28a^39^ and a strong expression of dendritic cell markers CD209 (DC-SIGN), a receptor involved in pathogen recognition and immune response initiation,^13^ as well as CD11c, an integrin characteristic of dendritic cells, which is associated with cell adhesion and migration.^40^ The differentiated cells also expressed CD83,^41^ a marker indicative of dendritic cell maturation, and CD86, a co-stimulatory molecule essential for T-cell activation through interaction with CD28.^42^ Flow cytometry additionally demonstrated high surface marker expression of Human Leucocyte Antigen-DR (HLA-DR), a subset of Major Histocompatibility Complex class II (MHC class II) molecules, which is crucial for antigen presentation.^43^ This confirms that the iPSC-DC acquired key functional characteristics associated with antigen-presenting cells, indicating successful differentiation.

The literature describes a wide variety of dendritic cell subsets with distinct phenotypes and functions depending on their origin and environmental context.^13,43^ While it is acknowledged that iPSC-derived dendritic cells may not fully correspond to a homogeneous subset observed in vivo, their differentiation and functionality were confirmed through the use of established dendritic cell markers.^13,41,44,45^ This detailed characterization validates the generation of functional iPSC-DC from pluripotent stem cells.

The antigen-presenting capacity of iPSC-DC was demonstrated via a MLR assay, where significant T-cell proliferation validated their ability to activate immune responses through the activation of T-cells. These findings highlight the potential of iPSC-DC as a consistent and scalable alternative to primary dendritic cells, overcoming donor variability and availability constraints.

The development of the immunocompetent skin model represents a major step forward in dermatological and toxicological research. By co-culturing iPSC-FB, iPSC-KC, and iPSC-DC in a fully integrated system, the results of this study show that these immunocompetent skin models not only mimic the structural and functional complexity of native skin, but also possess immune competence, which is crucial for assessing skin sensitization responses.

Sphingolipids are critical for the structure and function of the skin barrier, making their accurate representation in skin models essential for physiological relevance. As key components of the epidermal lipid matrix in the stratum corneum, they maintain skin hydration, prevent transepidermal water loss, and provide a protective barrier against external insults. Beyond their structural role, sphingolipids regulate vital signaling pathways involved in keratinocyte differentiation, epidermal homeostasis, immune response, and inflammation. For instance, ceramides promote keratinocyte differentiation, while sphingosine and sphingosine 1-phosphate (S1P) modulate immune signaling and inflammatory processes, relevant to both healthy and diseased skin.^29^ Each cell type has its own individual sphingolipid profile, which makes it a valuable characteristic for cell type-specific analysis and characterization.^46^ Variations in sphingolipid composition can influence skin barrier permeability, resilience, and overall functionality, impacting studies on drug penetration, treatments for skin diseases, and lipid-related conditions such as atopic dermatitis and psoriasis.^29,30^ The close resemblance of sphingolipid profiles in iPSC-derived keratinocytes and fibroblasts to those of primary skin cells reinforces their suitability for constructing physiologically relevant immunocompetent skin models. These findings support the use of iPSC-derived cells in developing accurate models for research and therapeutic applications.

The presence of tight junction proteins such as claudin in the epidermal layer underlines the ability of the model to maintain a regulated barrier, which is essential to mimic in vivo skin conditions. The expression of filaggrin in the stratum corneum supports the integrity and barrier function of the epidermis, a key element in assessing the skin’s defense against external agents, including potential sensitizers.

The confirmation of CK10 and CK14 in the suprabasal and basal layers, respectively, further supports the successful differentiation of iPSC-KC into a stratified epithelium, recapitulating the organization found in native human skin.^20,47,48^ Despite these structural similarities, minor variations in epidermal thickness were observed, which are consistent with previous findings on iPSC-derived skin models. It was reported that hair follicle-derived skin models often exhibit a less organized epidermal structure compared to foreskin-derived models,^20^ which aligns with our observations that iPSC-KC display structural differences compared to primary keratinocytes. Similarly, it was found that while iPSC-derived skin models successfully mimic human skin architecture, the epidermal layers may not be as highly organized as in native tissue.^19^ These differences likely stem from variations in differentiation potential and the organizational capacity of iPSC-derived cells. The identification of involucrin in the outer epidermal layers reinforces barrier functionality, indicating an advanced stage of keratinocyte maturation that contributes to the formation of the cornified layer of the epidermis and protection against environmental insults.^49^ Vimentin expression was also observed in both the dermal and epidermal layers, further highlighting the model’s structural integrity. The characterization of the dermal and epidermal layer of the skin model is consistent with features observed in RHS models derived from NHEK and NHDF as well as HFDK and HFDF.^20,47,48^ Notably, the use of iPSC-FB in the immunocompetent skin model resulted in significantly reduced gel contraction compared to fibroblasts derived from hair follicles or human foreskin. This difference may be attributed to variations in ECM remodeling capacity, potentially linked to reduced expression or activity of contractile proteins such as α-SMA or MMPs. Further investigations are necessary to elucidate the underlying mechanisms. In addition, we assessed intra- and inter-batch variability to determine the reproducibility of the epidermal structure. While some degree of variation in epidermal thickness was noted, the overall morphology and immune competence of the model remained consistent across batches. Future refinements, including optimization of cell seeding protocols, differentiation conditions, and culture media composition, may further improve epidermal uniformity and reproducibility.

A key finding of this study is the integration and activity of iPSC-DC directly within the skin model, a notable advancement over currently available skin models. Most published skin models rely on a co-culture setup, where monocyte-derived dendritic cells (MoDC) or monocyte-derived Langerhans cells (MoLC) are not fully integrated into the three-dimensional structure but are instead cultured within a gel and layered between the dermis and epidermis at a later stage of the model’s assembly.^26,28^ Several co-culture models have been developed to mimic immune responses in reconstructed human epidermis. Schellenberger et al.^27^ and Eskes et al.^50^ described models combining an epidermal equivalent with THP-1 cells, a widely used monocyte-derived cell line. These models enabled the assessment of immune activation and cytokine responses following exposure to sensitizers. For the COCAT assay (co-culture of HaCat and THP-1 cells) described by Eskes et al.,^50^ the cells were only co-cultured for 24 h, and no fibroblasts were included in the system. Similarly, in the THP-1 co-culture system with a 3D reconstructed epidermis, described by Schellenberger et al.,^27^ the co-culture also lasted only 24 h, and no fibroblasts were present. Moreover, in this model there is no direct integration of THP-1 cells within the epidermis, probably due to an activation without sensitizing molecules. Therefore, THP-1 cells were seeded in the basolateral compartment underneath the reconstructed human epidermis (RHE) models rather than being directly integrated within the epidermis. This setup may limit direct interactions between immune cells and keratinocytes, affecting their ability to recapitulate the complex immune dynamics of native skin.

To our knowledge, this is the first iPSC-derived immunocompetent skin model that successfully co-cultured iPSC-derived fibroblasts, keratinocytes, and dendritic cells in a fully integrated system. Similar approaches have been explored in other studies. Bock et al.^14^ incorporated MUTZ-LC into the epidermal layer of a reconstructed skin model, while Hölken et al.^22^ integrated THP-1 cells into the epidermal layer. Additionally, Böttcher et al.^25^ developed a full-thickness skin model with successfully incorporated MUTZ-LC as surrogates for dendritic cells, further demonstrating the potential of immune cell integration in 3D skin constructs. The concept of immunocompetent skin models with incorporated MUTZ-LC was first introduced by Kosten et al.,^23^ who successfully integrated these Langerhans-like cells into a reconstructed human skin equivalent, providing an early demonstration of immune cell functionality within a 3D skin model. These studies have contributed significantly to the advancement of immunocompetent skin models, supporting the relevance and applicability of our approach in improving physiological accuracy and immune responsiveness in in vitro skin sensitization models.

The baseline expression of CD86 in the dendritic cells distributed within the epidermal layer highlights the ability of iPSC-DC to participate in T-cell activation,^13,41,51,52^ a critical step in the immune response during skin sensitization. Additionally, the presence of dectin-1 demonstrates the model’s capacity to recognize and respond to sensitizing agents.^13^ These findings confirm that the integrated dendritic cells replicate key immune events, including antigen presentation and downstream signaling, within the context of skin sensitization.

This study further demonstrates the capacity of the iPSC-derived immunocompetent skin model to assess cytokine-mediated responses during skin sensitization. By targeting cytokines associated with key event 2 (keratinocyte activation) and maturation of iPSC-DC in key event 3 (dendritic cell activation) within the AOP for skin sensitization,^51,53^ the findings provide valuable insights into the model’s ability to replicate critical immune processes.

The selected cytokines play pivotal roles in inflammatory and immune responses during skin sensitization. IL-8, secreted by keratinocytes and dendritic cells, bridges innate and adaptive immunity through neutrophil recruitment.^10^ IL-1β amplifies inflammation while promoting keratinocyte activation and dendritic cell maturation.^54,55^ MIP-1β, predominantly secreted from dendritic cells, recruits immune cells and supports T-cell interactions,^11^ while IL-18 enhances T-cell activation and inflammatory signaling.^12^ TGF-β contributes to immune regulation and tissue remodeling,^56^ while keratinocyte-secreted MMP-9 aids extracellular matrix remodeling and immune cell migration.^57^ Finally, TSLP links keratinocyte signaling to dendritic cell activation, connecting key event 2 and key event 3.^58^ The results align closely with the AOP for skin sensitization. Significant upregulation of IL-8, IL-1β, MIP-1β, and TGF-β in response to extreme (DNCB, *p*-phenylenediamine) and moderate (isoeugenol) sensitizers confirms the activation of keratinocytes and dendritic cells. Elevated MMP-9 and TSLP secretion further support key event 2, while robust IL-18 and MIP-1β responses validate key event 3.

The model effectively differentiated substances based on their sensitization potential. Extreme sensitizers such as DNCB and *p*-phenylenediamine elicited the strongest cytokine secretion and dendritic cell maturation responses, including significantly elevated CD86, CD209, and HLA-DR expression after 24-h exposure, validating their high potency. The moderate sensitizers isoeugenol induced measurable but lower responses, while the weak sensitizer resorcinol demonstrated limited cytokine secretion, yet still induced a significant maturation activity. Non-sensitizing compounds like glycerol induced no significant changes in maturation or cytokine secretion, confirming their lack of sensitization potential. The interconnected roles of cytokines, particularly IL-8 and IL-1β in key event 2 and key event 3, emphasize the model’s physiological relevance and alignment with the AOP framework. Maturation assessments further validate the model’s ability to replicate DC-mediated immune activation, providing comprehensive insights into the molecular and cellular events underlying sensitization. These readouts have been widely accepted in the literature and have been successfully utilized in several publications,^14,17,22,28^ demonstrating their robustness and reproducibility in modeling key events in skin sensitization. Importantly, the immunocompetent skin model is capable of detecting not only extreme sensitizers, but also weak sensitizers, highlighting its sensitivity and broad applicability in assessing a wide range of sensitizing compounds.

The thorough characterization of surface markers underscores the robustness of the iPSC-derived immunocompetent skin model, providing a strong foundation for its use in studies of immune responses and skin sensitization. This iPSC-derived skin model represents a scalable, physiologically relevant tool for studying sensitization mechanisms and regulatory applications. By addressing key event 2 and key event 3, it offers a robust platform for identifying and characterizing skin sensitizers, advancing preclinical testing while supporting the shift toward ethical, predictive alternatives in skin research.

This finding is particularly relevant for evaluating the sensitizing potential of chemical and cosmetic products. By fully integrating dendritic cells into the model, the interplay between skin cells and immune cells is effectively recapitulated, creating a more physiologically relevant platform. This advancement offers a promising in vitro alternative for assessing sensitizing compounds, meeting the growing demand for reliable, ethical, and non-animal testing methods while pushing the boundaries of skin model research.

Immunocompetent skin models offer a distinct advantage in their ability to evaluate complete formulations, a capability not feasible with in silico methods. This broadens their application in preclinical research, particularly for complex chemical mixtures and formulations. Unlike in chemico assays, which primarily rely on the structural reactivity of a substance, immunocompetent skin models are metabolically active.^59^ This metabolic capacity enables the detection and evaluation of pre-haptens and pro-haptens, substances requiring metabolic activation to exhibit reactivity, overcoming a significant limitation of in chemico methods.^59,60^ This mechanistic relevance underscores the suitability of immunocompetent models for a comprehensive assessment of skin sensitization potential. To our knowledge, this is the first iPSC-derived immunocompetent skin model capable of responding to skin sensitizers of varying potencies, representing a significant step forward in the development of advanced, physiologically relevant testing platforms. Unlike conventional assays such as DPRA, KeratinoSens™, and h-CLAT, which assess only isolated key events, the immunocompetent skin model allows for simultaneous evaluation of keratinocyte activation (key event 2) and dendritic cell activation (key event 3). Compared to Sens-IS and IL-18 assays, which rely on cytokine profiling from keratinocytes alone, the immunocompetent skin model incorporates fully integrated iPSC-DC, enabling direct antigen presentation and a more dynamic immune response assessment. Furthermore, it provides a functional epidermal barrier with tight junctions and sphingolipids, further enhancing physiological relevance. This ability to replicate key immune mechanisms positions our model as a promising alternative to existing Defined Approaches.

Additionally, the immunocompetent skin model shows strong potential for integration into commercial testing systems due to its high reproducibility and scalability. A major advantage of this model is that iPSC-FB and iPSC-KC only need to be generated once and can be cryopreserved for long-term use, ensuring consistent and reproducible results. Only iPSC-DC require regular generation, significantly reducing donor variability compared to conventional skin models that rely on primary cells. This reduction in variability, combined with its ability to detect weak sensitizers more effectively than Sens-IS and IL-18 assays, highlights its robustness in skin sensitization assessments. Furthermore, this model allows for the generation of individualized skin models, offering opportunities for personalized testing and precision medicine. These features collectively enhance the model’s practical applicability for routine safety assessments and commercial testing, aligning with regulatory demands for more reliable and human-relevant in vitro models.

## Supplemental Material

sj-docx-1-tej-10.1177_20417314251336296 – Supplemental material for Development of an iPSC-derived immunocompetent skin model for identification of skin sensitizing substances Supplemental material, sj-docx-1-tej-10.1177_20417314251336296 for Development of an iPSC-derived immunocompetent skin model for identification of skin sensitizing substances by Marla Dubau, Tarada Tripetchr, Lava Mahmoud, Fabian Schumacher and Burkhard Kleuser in Journal of Tissue Engineering

sj-tiff-2-tej-10.1177_20417314251336296 – Supplemental material for Development of an iPSC-derived immunocompetent skin model for identification of skin sensitizing substances Supplemental material, sj-tiff-2-tej-10.1177_20417314251336296 for Development of an iPSC-derived immunocompetent skin model for identification of skin sensitizing substances by Marla Dubau, Tarada Tripetchr, Lava Mahmoud, Fabian Schumacher and Burkhard Kleuser in Journal of Tissue Engineering

sj-tiff-3-tej-10.1177_20417314251336296 – Supplemental material for Development of an iPSC-derived immunocompetent skin model for identification of skin sensitizing substances Supplemental material, sj-tiff-3-tej-10.1177_20417314251336296 for Development of an iPSC-derived immunocompetent skin model for identification of skin sensitizing substances by Marla Dubau, Tarada Tripetchr, Lava Mahmoud, Fabian Schumacher and Burkhard Kleuser in Journal of Tissue Engineering
