# Supplementary material for: Development of an iPSC-derived immunocompetent skin model for identification of skin sensitizing substances
Source: J Tissue Eng. 2025 May 6;16:20417314251336296. doi: 10.1177/20417314251336296 (PMC12056326; doi:10.1177/20417314251336296)
Supplement: sj-docx-1-tej-10.1177_20417314251336296 – Supplemental material for Development of an iPSC-derived immunocompetent skin model for identification of skin sensitizing substances [file sj-docx-1-tej-10.1177_20417314251336296.docx]

**Supplemental material for article:** **Development of an iPSC-derived immunocompetent skin model for *in vitro* identification of skin sensitizing substances**


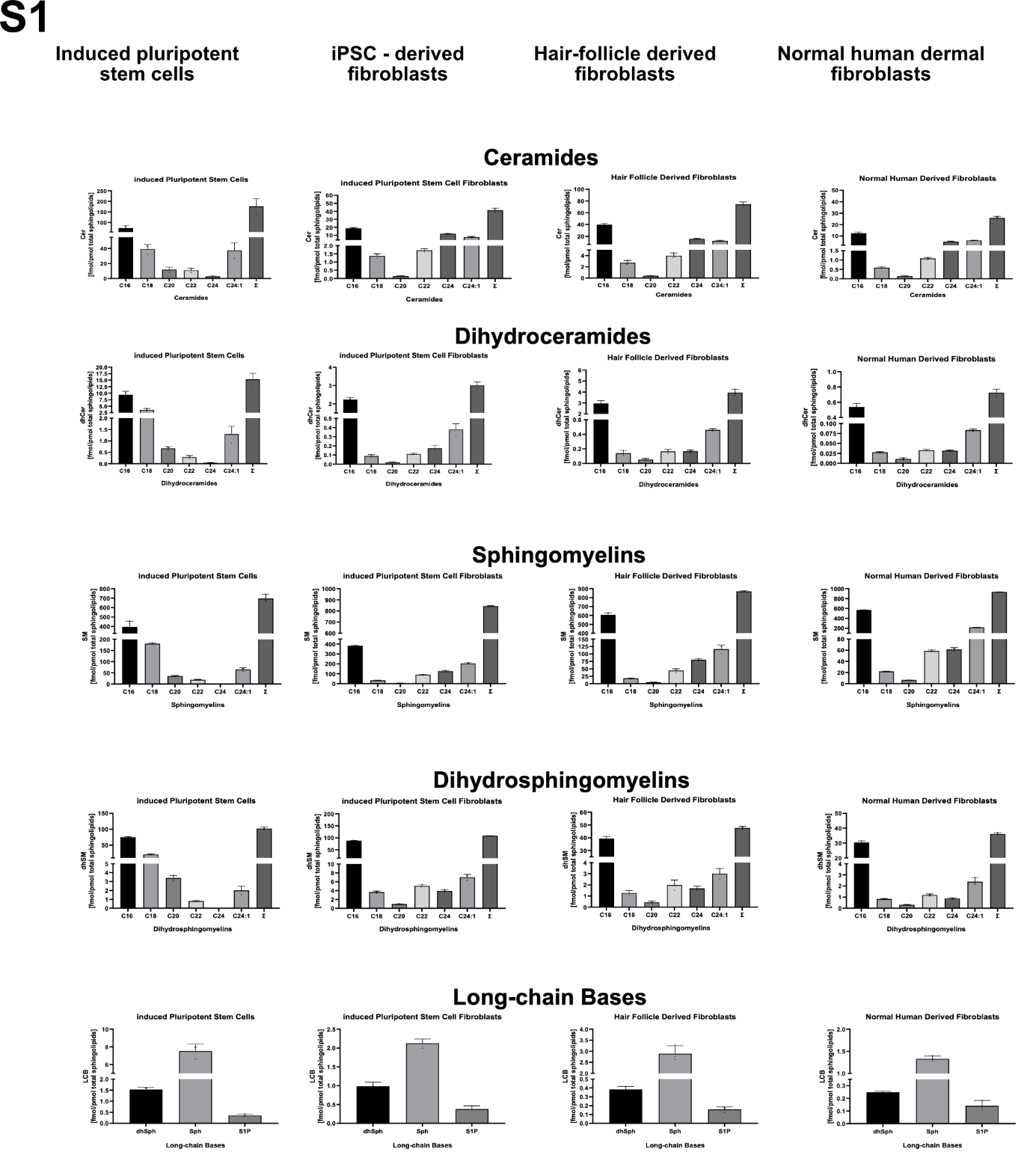


Supplementary figure 1. Quantitative analysis of sphingolipids in iPSC in comparison to hair-follicle, normal human foreskin-derived and iPSC-derived fibroblasts determined by LC-MS/MS. Levels of sphingolipid species, including ceramides, dihydroceramides, sphingomyelins, dihydrosphingomyelins, and long-chain bases, were quantified in iPSC-FB and compared to HFDF, NHDF and undifferentiated iPSC. Shown is the mean ± SD of 3 independent experiments.


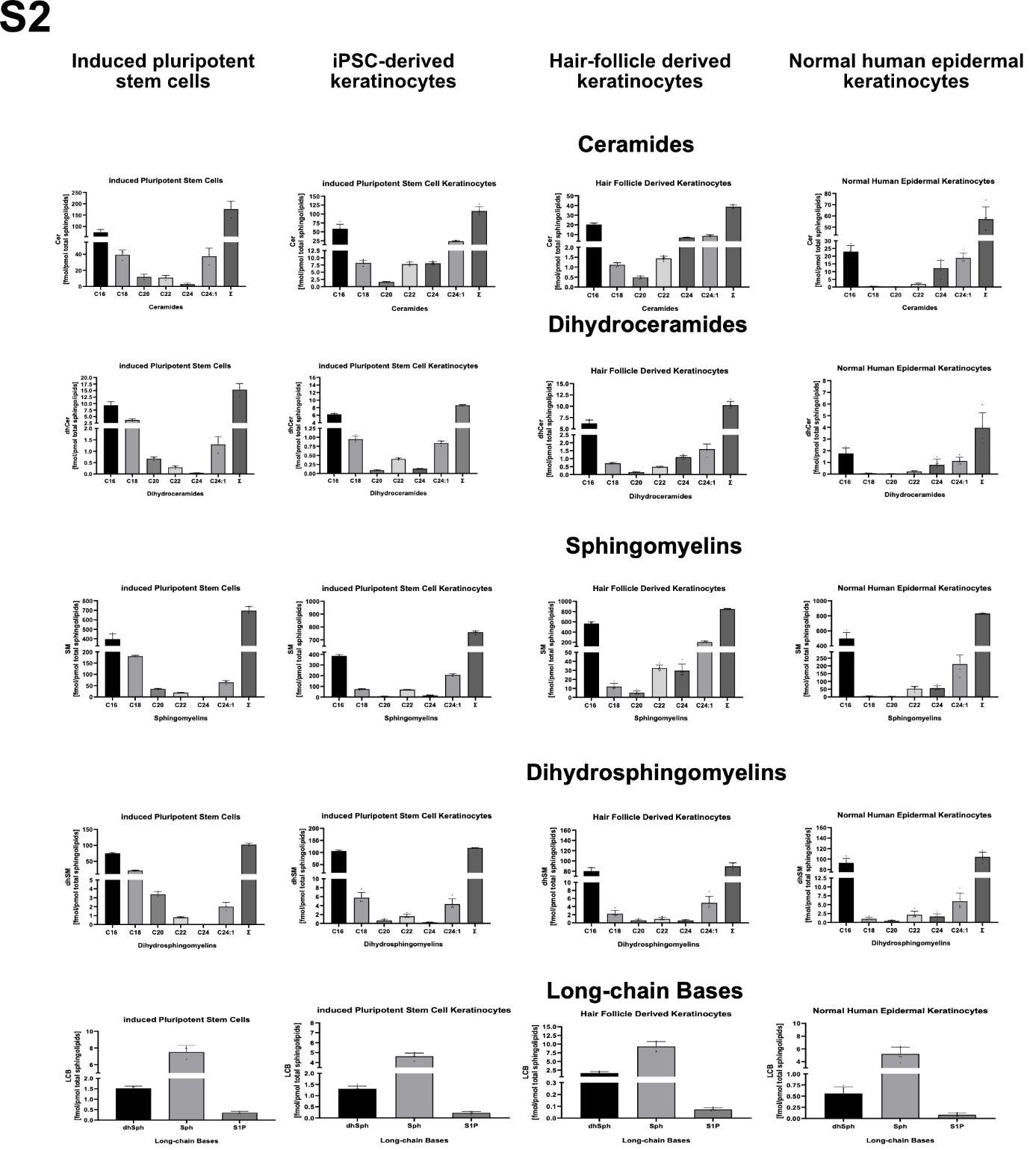


Supplementary figure 2. Quantitative analysis of sphingolipids in iPSC in comparison to hair-follicle, normal human foreskin-derived and iPSC-derived keratinocytes determined by LC-MS/MS. Levels of sphingolipid species, including ceramides, dihydroceramides, sphingomyelins, dihydrosphingomyelins, and long-chain bases, were quantified in iPSC-KC and compared to HFDK, NHEK and undifferentiated iPSC. Shown is the mean ± SD of 3 independent experiments.
