# Supplementary figures and images for: Development of an iPSC-derived immunocompetent skin model for identification of skin sensitizing substances
Source: J Tissue Eng. 2025 May 6;16:20417314251336296. doi: 10.1177/20417314251336296 (PMC12056326; doi:10.1177/20417314251336296)

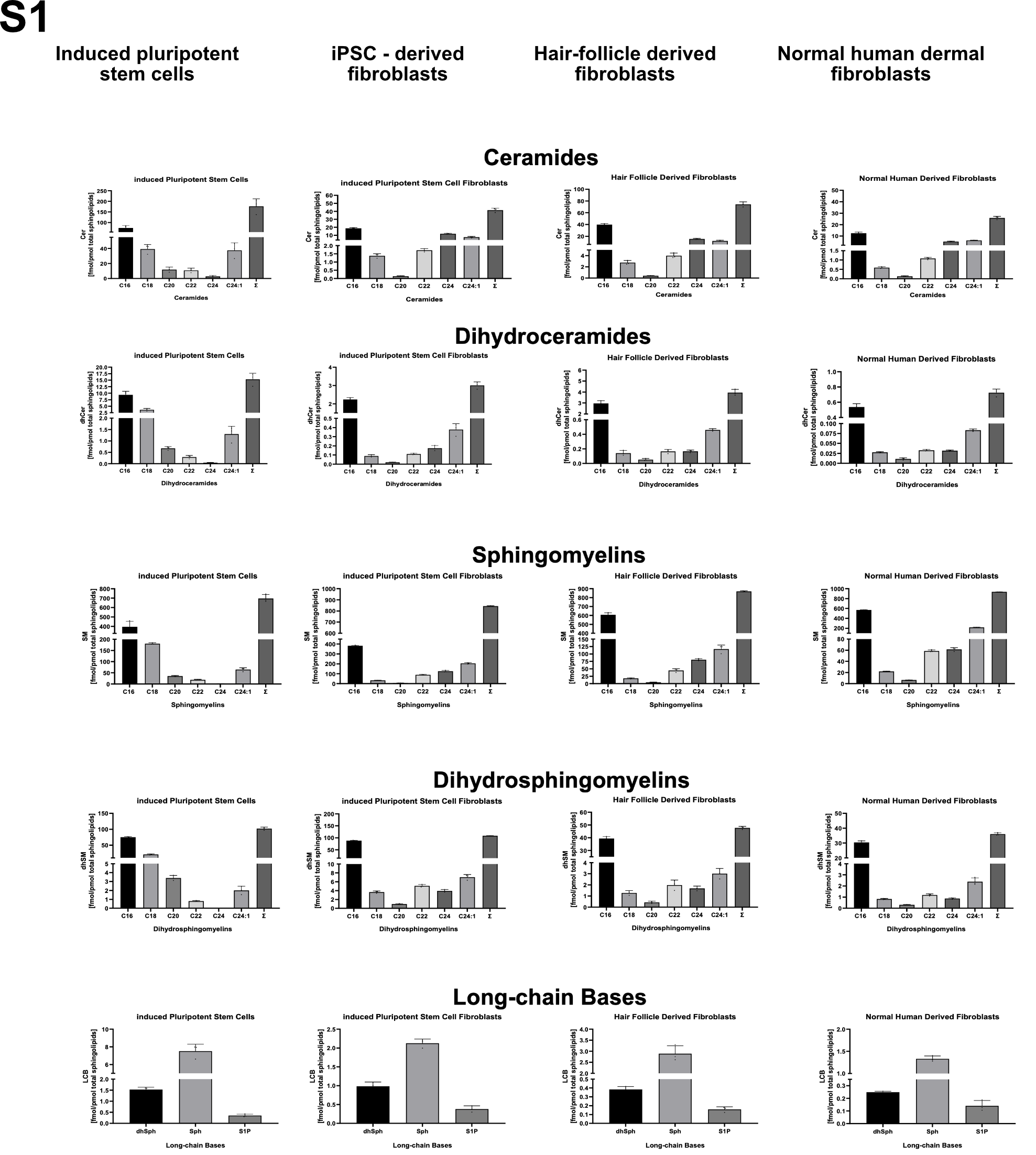

Supplement: sj-tiff-2-tej-10.1177_20417314251336296 – Supplemental material for Development of an iPSC-derived immunocompetent skin model for identification of skin sensitizing substances [file sj-tiff-2-tej-10.1177_20417314251336296.tiff]

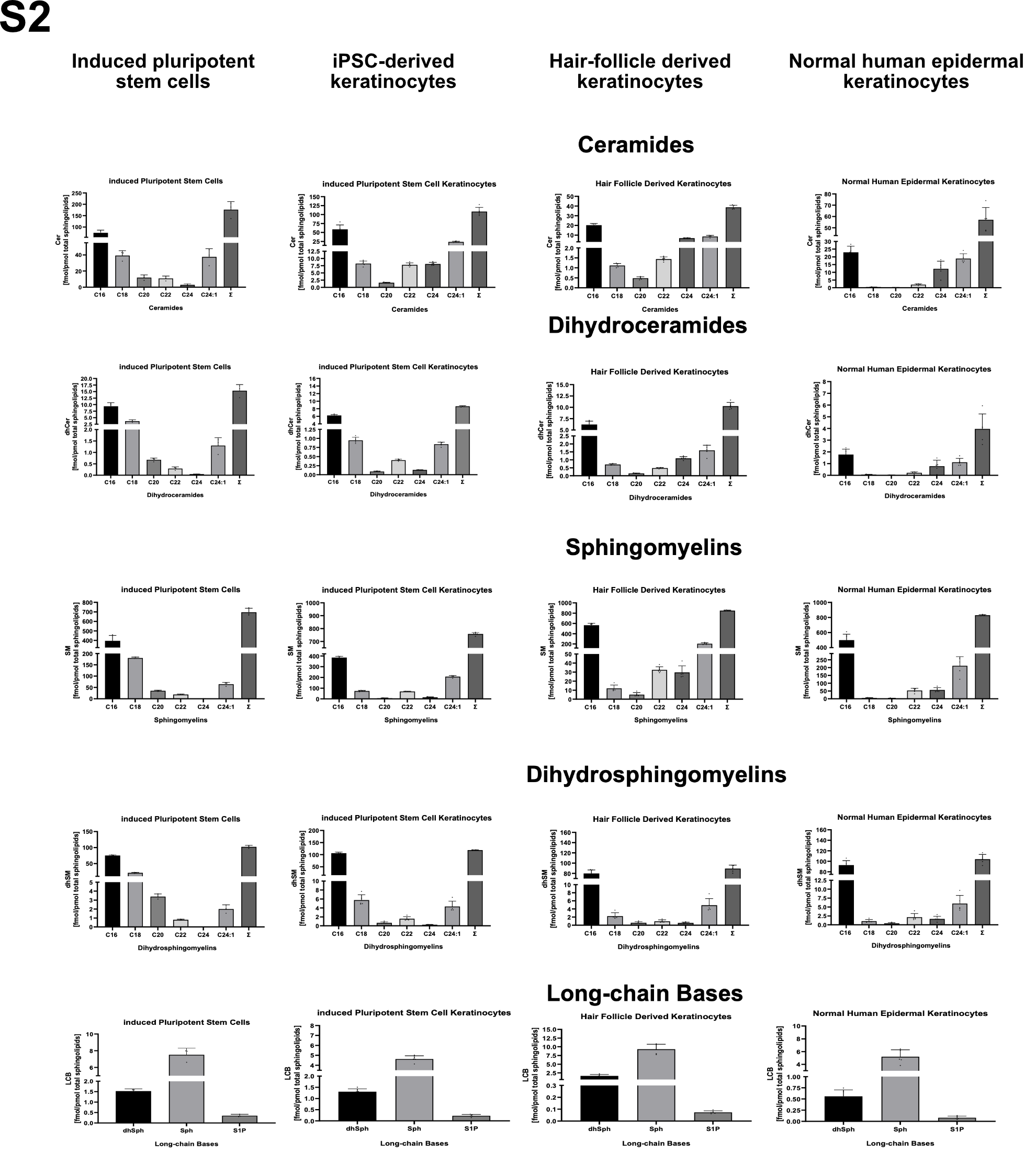

Supplement: sj-tiff-3-tej-10.1177_20417314251336296 – Supplemental material for Development of an iPSC-derived immunocompetent skin model for identification of skin sensitizing substances [file sj-tiff-3-tej-10.1177_20417314251336296.tiff]
